# Mitigation of water scarcity with sustained growth of Rice by plant growth promoting bacteria

**DOI:** 10.3389/fpls.2023.1081537

**Published:** 2023-01-23

**Authors:** Naima Mahreen, Sumera Yasmin, Muhammad Asif, Mahreen Yahya, Khansa Ejaz, Mehboob-ur-Rahman, Sumaira Yousaf, Imran Amin, Sana Zulfiqar, Asma Imran, Shazia Khaliq, Muhammad Arif

**Affiliations:** ^1^ Soil and Environmental Biotechnology Division, National Institute for Biotechnology and Genetic Engineering College, Pakistan Institute for Engineering and Applied Sciences (NIBGE-C, PIEAS), Faisalabad, Punjab, Pakistan; ^2^ Agricultural Biotechnology Division, National Institute for Biotechnology and Genetic Engineering College, Pakistan Institute for Engineering and Applied Sciences (NIBGE-C, PIEAS), Faisalabad, Punjab, Pakistan; ^3^ Nuclear Institute for Agriculture and Biology (NIAB) College, Pakistan Institute for Engineering and Applied Sciences (NIAB-C, PIEAS), Faisalabad, Punjab, Pakistan; ^4^ Industrial Biotechnology Division, National Institute for Biotechnology and Genetic Engineering College, Pakistan Institute for Engineering and Applied Sciences (NIBGE-C, PIEAS), Faisalabad, Punjab, Pakistan

**Keywords:** sustainable agriculture, water scarcity, abiotic stress, Super Basmati, drought tolerant bacteria, IRGA, infrared thermal (IRT) imager, florescent *in situ* hybridization

## Abstract

Climate change augments the risk to food security by inducing drought stress and a drastic decline in global rice production. Plant growth-promoting bacteria (PGPB) have been known to improve plant growth under drought stress. Here in the present study, we isolated, identified, and well-characterized eight drought-tolerant bacteria from the rice rhizosphere that are tolerant to 20% PEG-8000. These strains exhibited multiple plant growth-promoting traits, i.e., 1-aminocyclopropane-1-carboxylic acid (ACC) deaminase activity, exopolysaccharide production, phosphate (P)-solubilizing activity (51–356 µg ml^-1^), indole-3 acetic acid (IAA) production (14.3–46.2 µg ml^-1^), and production of organic acids (72–178 µg ml^-1^). Inoculation of bacterial consortium (*Bacillus subtilis* NM-2, *Brucella haematophilum* NM-4, and *Bacillus cereus* NM-6) significantly improved seedling growth and vigor index (1009.2-1100) as compared to non-inoculated stressed plants (630-957). Through rhizoscanning, efficiency of the consortium was validated by improved root parameters such as root length (17%), diameter, and surface area (18%) of all tested genotypes as compared with respective non-inoculated stressed treatments. Furthermore, the response of consortium inoculation on three rice genotypes was positively correlated with improved plant growth and drought stress ameliorating traits by the accumulation of osmoprotectant, i.e., proline (85.8%–122%), relative water content (51%), membrane stability index (64%), and production of antioxidant enzymes to reduce oxidative damage by reactive oxygen species. A decrease in temperature and improved chlorophyll content of inoculated plants were found using infrared thermal imaging and soil plant analyzer development (SPAD), respectively. The key supporting role of inoculation toward stress responses was validated using robust techniques like infrared thermal imaging and an infrared gas analyzer. Furthermore, principal component analysis depicts the contribution of inoculation on stress responses and yield of tested rice genotypes under water stress. The integration of drought-tolerant rice genotype (NIBGE-DT02) and potential bacterial strains, i.e., NM-2, NM-4, and NM-6, can serve as an effective bioinoculant to cope with water scarcity under current alarming issues related to food security in fluctuating climate.

## 1 Introduction

Climate change and unfavorable abiotic factors in the recent era have adversely affected crop productivity and economic yields from agricultural land. Water scarcity, severe drought, and temperature extremes are reducing moisture levels in soil profiles, altering plant physiology, and reducing growth and yields (Deng et al., 2021). The worst heatwave due to global warming hit the water supply under threat while China and Europe face severe droughts in recent years (JRC, 2022; Global times, 2022). According to the United Nations Global Land Outlook report 2020–2022, Pakistan is one of 23 countries that have faced emergencies related to drought in the last 2 years (UNCCD, 2022).

Rice (*Oryza sativa* L.) as a paddy field crop is particularly susceptible to water scarcity. Due to insufficient, uneven, and unpredictable rainfall during the growing season, it is estimated that 50% of the world′s rice production will be decreased dramatically (Al-Zahrani et al., 2022). To alleviate global food insecurity, a large number of rice irrigations have to be replaced by different management strategies (Aalipour et al., 2020). Currently, strategies to enhance the plant′s ability to cope with drought stress include traditional breeding, genetic engineering of drought-tolerant transgenic plants, and the use of water-saving irrigation. Unfortunately, these strategies are very complex, time-consuming, and labor-intensive. Therefore, we need quick, reliable *in situ* methods that can be widely applied to enhance the ability of plants to produce more under limited irrigations (Kannepalli et al., 2020).

The use of plant growth-promoting bacteria (PGPB) is an effective approach to grow plants under water scarcity. Directly or indirectly, they can promote plant growth under biotic or abiotic stress environments (Fadiji et al., 2022). Known mechanisms of PGPB to improve plant growth and its survival in stressed environments, especially under drought conditions, include phytohormone production (IAA), phosphate (P) solubilization, exopolysaccharide (EPS) production, and siderophore production for sequestration of iron (Ilyas et al., 2020). In addition, PGPB is associated with the molecule′s catabolism, i.e., bacterial 1-aminocyclopropane-1-carboxylate (ACC) deaminase production involved in signaling under stress. Ethylene content in plants is usually increased in stress; thus, inoculation with ACC deaminase-producing bacteria mitigates the effect of osmotic stress in plants by reducing the production of ethylene (Sagar et al., 2020).

Polyethylene glycol (PEG)-induced drought stress decreased the seed germination, vigor index, and seedling growth of rice as compared with well-irrigated seedlings (Delshadi et al., 2017). Most of the studies reported that inoculation of phosphate-solubilizing and indole acetic acid (IAA)-, 1-aminocyclopropane-1-carboxylate (ACC)-, and exopolysaccharide (EPS)-producing bacteria significantly improved percent seed germination and seedling vigor index of different plants, i.e., wheat, grapevine, and tomato under osmotic stress (Ilyas et al., 2020; Duan et al., 2021; Khairnar et al., 2022). PGPB are an important biological element with beneficial effects on plant biochemical and physiological properties, resulting in improved photosynthetic pigments, osmolyte production, leaf number, and relative water content (Fadiji et al., 2022). Wheat, maize, and rice plants inoculated with PGPB resulted in an improved root/shoot length, fresh/dry weights, leaf chlorophyll, membrane stability index (MSI), proline content, and upregulation in the antioxidants such as peroxidase (POD), catalase (CAT), superoxide dismutase (SOD), polyphenol oxidase (PPO), and phenylalanine (PAL) to scavenge the production of reactive oxygen species (ROS) under drought conditions (Mathur et al., 2018; Li X et al., 2020; Inbaraj, 2021).

Solute accumulation is one of the most important mechanisms adopted by plants to withstand drought stress. PGPB confer tolerance to drought stress in plants by improving the plant′s ability to produce osmoprotectants like proline (Ngumbi and Kloepper, 2016). Bacterial communities not only alter the hormonal balance of the roots but also improve plant nutrient and water uptake, resulting in microbial colonization and symbiotic relationships (Nemeskeri et al., 2022). Nemeskeri et al. (2019) reported that an improvement in plant water uptake also reduced the plant temperature as compared with non-inoculated stressed plants as high plant temperature has an adverse impact on yield. Infrared thermography (IRTI) has appeared as an easy-to-use, non-destructive, and promising technique for measuring plant physiological status related to water availability (Kim et al., 2020).

Drought stress reduces the rice plant′s yield, whereas PGPB inoculation improved the plant traits such as height, panicles, plant weight, and yield grown even under drought stress (El-Mageed et al., 2022).

Overall, drought-tolerant rhizobacteria offer an eco-friendly and efficient approach to alleviate plant stress and enhance plant growth and yield under adverse climatic conditions. Therefore, in the present study, we focused on i) isolating, screening, and selecting drought-tolerant rhizobacteria and ii) evaluating efficient rhizobacteria for rice growth promotion under water stress conditions through the study of morpho-physiological and biochemical parameters. We hypothesized that i) drought-tolerant bacteria with plant growth promoting (PGP) traits can improve plant vigor and root architecture through phytohormone (IAA), osmolyte, and exopolysaccharide production, which ultimately increases the yield of rice under water stress conditions, and ii) integrated application of potential drought-tolerant bacteria obtained in this study and advanced non-destructive *in situ* handheld instruments, i.e., infrared thermal imaging (IRTI) and infrared gas analyzer (IRGA), with conventional methods may lead to the efficient selection and timely intervention of a tolerant variety to minimize yield loss from water scarcity. This integration of biological strategy with high-quality imagery and physiological processes will lead to a useful day-by-day comparison for the detection of drought stress, which is the need of the hour because conventional methods require a long time for the detection of drought conditions.

## 2 Materials and methods

### 2.1 Soil sample collection

Rice rhizosphere soil samples were collected from plants grown in Faisalabad (31°24′10.7″N 73°01′31.4″E), Sheikhupura (31°35′15.4″N 73°40′12.5″E), Gujranwala (32°09′29.7″N 74°01′28.3″E), Kala Shah Kaku (31°43′17.0″N 74°16′04.9″E), Sialkot (32°29′01.0″N 74°24′08.3″E), Hafizabad (32°08′59.4″N 73°29′06.4″E), Pindi Bhattian (31°54′09.0″N 73°15′35.0″E), Nankana Sahib (31°53′20.5″N 73°40′02.8″E), Narang Mandi (31°54′00.9″N 74°29′54.0″E), and Farooqabad (31°44′51.2″N 73°46′36.6″E). Soil samples were collected at a depth of 0–20 cm and were carried to the laboratory (Passari et al., 2017). Soil adhering to the roots was separated, homogenized, and stored at 4°C till further use for isolation of rhizosphere bacteria.

### 2.2 Isolation of rhizobacteria

The dilutions of soil samples up to 10^-4^ were prepared, and each dilution (100 µl) was spread on Luria Bertani (LB) agar petri plates. Then, plates were incubated for 24–48 h at 28 ± 2°C. Different morphotypes were identified and subcultured to get pure colonies of each morphotype (Murtaza et al., 2014).

### 2.3 Screening of rhizobacteria for drought tolerance

Pure colonies of each morphotype were screened for drought stress under different concentrations of PEG-8000 in an *in vitro* study. Drought stress of 5%, 10%, 15%, and 20% was developed gradually by dissolving 5, 10, 15, and 20 g of PEG-8000, respectively, in 100 ml LB broth medium while the non-stressed medium was prepared without PEG, i.e., 0% PEG (Michel and Kaufmann, 1973; Niu et al., 2018). Broth media containing 5% PEG concentration and non-stressed media were inoculated with overnight grown cultures (10^8^ CFU ml^-1^) and incubated for 2–3 days on the shaker at 28 ± 2°C. To determine the tolerance of bacterial strains to the specific level of drought stress created by PEG-8000, the growth was determined by measuring optical density (OD) on a spectrophotometer (CamSpec UV-Vis M550, UK) at 600 nm and by viable count through the spread plate method (Somasegaran and Hoben, 1994).

### 2.4 Bioassay of drought-tolerant rhizobacteria for PGP traits

#### 2.4.1 Indole-3 acetic acid production

The detection of IAA production was carried out by the colorimetric method (Gordon and Weber, 1951). For this, fresh bacterial colonies were grown in LB broth containing 0.01% tryptophan for 7 days. The production of IAA was detected by mixing 100 µl of culture with 100 µl of Salkowski’s reagent in a titer plate. After incubation for 30 min at room temperature, the appearance of pink color was an indication of IAA production. Further, IAA was quantified by treating the culture supernatant with ethyl acetate. The extract was evaporated to dryness collected with methanol and subjected to high-performance liquid chromatography (HPLC) (Perkin Elmer) accompanied by a column (C-18) at 0.5 ml min^-1^ flow rate (Tien et al., 1979). Production of IAA was determined by retention time and peak area of 1000 ppm synthetic IAA standard.

#### 2.4.2 Phosphate solubilization and detection of organic acids produced

The phosphate solubilization potential of bacteria was checked on National Botanical Research Institute’s Phosphates (NBRIP) agar medium supplemented with tri-calcium phosphate (TCP) as a P source (Nautiyal, 1999). The bacteria were cultured on the petri plates that were kept at 28 ± 2°C in an incubator for 7–14 days. Formation of the halo zone around the colonies was considered positive for phosphate solubilization. The solubilization index of P was measured with the formula described by Shanware et al. (2014). Quantification of P solubilization by the bacteria was determined in NBRIP broth medium by the molybdate blue method using a spectrophotometer (CamSpec UV-Vis M550, UK) at 882 nm (Murphy and Riley, 1962). Different concentrations of standard solution (KH_2_PO_4_) were used for the standard curve, and tested samples were compared for P quantification (Soltanpour and Workman, 1979).

Different organic acids produced by bacteria *in vitro* during P solubilization were detected on HPLC (Varian ProStar 325 UV-Vis, United States) by using inoculated NBRIP filtrate (Vyas and Gulati, 2009). The control was filtrate of a non-inoculated NBRIP broth. The retention time and peak of standard solutions (100 µg l^-1^) were compared with tested samples for organic acid quantification (Macias-Benitez et al., 2020).

#### 2.4.3 Exopolysaccharide production

Exopolysaccharide production was determined by growing bacterial isolates in a 20% sucrose-supplemented GYC broth medium containing 50 g of glucose, yeast extract (10 g), and calcium carbonate at 5 g l^-1^. The cultures were then centrifuged at 6,000 rpm for 10 min, and the supernatant was collected. Trichloroacetic acid (TCA: 14% w/v) was added to the supernatant to precipitate the proteins in the polysaccharides. Flasks were then placed on a shaking incubator at 50 rpm and 23°C for 40 min followed by centrifugation at 10,000 rpm and 4°C for 10 min. Then, ethanol precipitation was done to purify EPS according to the method described by Anwar et al. (2010). A thin-layer chromatography (TLC) plate (Silica gel 60 F254; Merck) was spotted with 2 µl of supernatant and run for 6 h in the mobile phase (butanol 5: ethanol 5: water 3). An air-dried TLC plate was sprayed with urea-developing solution followed by heating for 15 min at 120°C to detect the fructose-containing compounds as described by Nasir et al. (2020). The retention factor (R_f_) of the samples was measured by the following formula:


$$Rf=\frac{Distance\;travelled\;by\;the\;substance}{Distance\;travelled\;by\;the\;solvent}$$

#### 2.4.4 Siderophore production

Production of siderophores by the bacteria was tested by the method described by Ames-Gottfred et al. (1989). Chrome Azurol S (CAS) agar plates were streaked with pure bacterial colonies and incubated at 28 ± 2°C for 3–4 days. The plates were observed for orange or purple coloration that appeared around bacterial culture as described by Ali et al. (2014).

#### 2.4.5 ACC-deaminase activity

The potential of bacteria to break down ACC was determined using 0.5 M ACC containing DF (Dworkin and Foster) minimal salt medium (Penrose and Glick, 2003). The inoculated bacteria were grown for 48–72 h at 28 ± 2°C with shaking at 13,000 rpm. The non-inoculated medium was considered as a control. Turbidity appeared in the medium and showed ACC-deaminase activity as described by Zia et al. (2021).

### 2.5 Identification of drought-tolerant PGP bacteria

The selected drought-tolerant plant growth-promoting bacteria were studied for colony morphology, gram staining, and cell motility using light microscopy following Bergey’s Manual (Vos et al., 2011).

Molecular identification of each drought-tolerant plant growth-promoting bacterium was carried out by the amplification of 16S rRNA gene using forward primer fD1 5′-AGAGTTTGATCCTGGCTCAG-3′ and reverse primer rD1 5′-AAGGAGGTGATCCAGCC-3′ as described by Imran et al. (2010). Agarose gel (1%) was used to analyze the amplified PCR products. The kit for PCR purification (QIAGEN Sciences, Germantown, USA) was used to purify the products before commercial sequencing from Macrogen (Macrogen, Inc., Seoul, South Korea). Sequences obtained were compared with gene sequences in the NCBI GenBank database using the BlastN algorithm. Then, nucleotide sequences were aligned and a phylogenetic tree was constructed using the method of maximum likelihood (Kumar et al., 2016). The sequences were submitted to NCBI GenBank (http://www.ncbi.nlm.nih.gov/).

### 2.6 Plant inoculation experiments to study the effect of drought-tolerant bacteria on rice under osmotic stress

#### 2.6.1 Development of drought tolerant plant growth-promoting consortium

A commercially available blood agar medium was used for the biosafety assessment of drought-tolerant PGP bacteria. Pure colonies were streaked on blood agar petri plates incubated at 28 ± 2°C and for 72 h as described by Mogrovejo et al. (2020). Zone formation around colonies was observed as indicative for hemolytic activity of the bacterium. An *in vitro* compatibility test of eight selected drought-tolerant bacteria (NM-1, NM-2, NM-3, NM-4, NM-5, NM-6, NM-7, and NM-8) was conducted for consortia development (Niu et al., 2018). The bacterial culture (10^8^ CFU ml^-1^, 100 µl) was spread on an LB agar plate against other tested strains as described by Yahya et al. (2021). Then, plates were incubated at 28 ± 2°C for 72 h. No colony formed the zone of inhibition, indicating that all the strains were compatible with each other.

Each bacterial culture (50 ml) of three strains, i.e., *Bacillus subtilis* NM-2, *Brucella haematophila* NM-4, and *Bacillus cereus* NM-6, grown separately in LB broth was mixed in an equal volume to prepare consortium (10^8^ CFU ml^-1^).

#### 2.6.2 Plant material used

Seeds of drought-susceptible rice genotype Super Basmati (Variety 1; V1) (Sabar and Arif, 2014; Mumtaz et al., 2019) and drought-tolerant rice genotypes NIBGE-DT02 (Variety 2; V2) and IR55419-04 (Variety 3; V3) (Sabar et al., 2019) selected in our previous study (Mahreen et al., 2022) were obtained from DNA Marker and Plant Genomics Lab of Agricultural Biotechnology Division (ABD), National Institute for Biotechnology and Genetic Engineering (NIBGE) Faisalabad.

#### 2.6.3 Plate assay and rhizoscanning for germination, seedling vigor index, and root morphology of rice seedlings

Based on the *in vitro* studies for drought tolerance and PGP traits, eight potential drought-tolerant isolates (NM-1, NM-2, NM-3, NM-4, NM-5, NM-6, NM-7, and NM-8) were selected to study their impact on seed germination and seedling vigor index (VI) separately and in a consortium.

For this assay, seed sterilization of rice genotypes was carried out with 3% NaOCl for 4–5 min and thrice washed with autoclaved H_2_O. Sterilized seeds of each genotype were soaked separately in the overnight grown culture of bacteria (1 × 10^8^ CFU ml^-1^) and in a consortium (1 × 10^8^ CFU ml^-1^) for 30 min. Seeds soaked in non-inoculated LB broth were considered as a control. This experiment was carried out in a growth room kept at 28°C for day and at 23°C for night with a light intensity of 460 µ mol m^-2^ s^-1^. Twenty seeds per petri plate were kept on moist filter paper at an equal distance in petri plates of 14-cm diameter. The plates were incubated at 28 ± 2°C for 72 h in the dark prior to shifting in the growth room. The experiment was conducted in a complete randomized design (CRD) with three biological replicates of four treatments, i.e., non-inoculated control (non-inoculated seeds + Hoagland solution), inoculated control (inoculated seeds + Hoagland solution), osmotic stressed (non-inoculated + 20% PEG-8000-supplemented Hoagland solution), and inoculated osmotic stressed (inoculated seeds + 20% PEG-8000-supplemented Hoagland solution) for each strain, and the experiment was repeated twice. Percent germination and different parameters related to seedling growth were measured at 7 days post inoculation (DPI) (Islam et al., 2016).

The effect of the most efficient consortium on root parameters of all the tested rice genotypes (IR55419, NIBGE-DT02, and Super Basmati) was studied using the Rhizo scanner (Model: EPSON, v700, CA, USA) accompanied by software WinRHIZO by Regent Instruments, BC, Canada. Three seedlings per replicate were selected to study root length, shoot length, and vigor index.

#### 2.6.4 Evaluation of drought-tolerant consortium on rice growth in a pot experiment under net house conditions

To study the inoculation effect of consortium (*Bacillus* sp. NM-2, *Brucella* sp. NM-4, and *Bacillus* sp. NM-6) on plant morpho-physiological and biochemical parameters, a pot experiment was carried out at NIBGE under net house conditions (31°24′10.7″N 73°01′31.4″E) in the rice growing season (July–October 2021). Three rice genotype (V1, V2, and V3) nurseries were sown. Recommended management practices for healthy seedlings were done as described by Zahid et al. (2020). Homogenized soil (pH 6.45, non-sterilized, N 4.3%, textured loamy, P 2%, and EC 3.1 mS cm^-1^) at 12 kg was filled in earthen pots of 14-inch height and 12-inch diameter. Before transplanting the rice nursery, earthen pots were saturated with water to settle down the soil for a few days. The soil level of pots was set aside at 5 cm below the edge. Prior to transplantation, the roots of rice seedlings were dipped in carrier material inoculated with the consortium (1 × 10^8^ CFU ml^-1^) for 30 min, whereas roots of control seedlings were dipped in non-inoculated LB broth for 30 min. The experiment was conducted in a randomized completely block design (RCBD) with three replicates of each treatment, i.e., non-inoculated control (well-irrigated and non-inoculated seedlings), inoculated control (well-irrigated and inoculated seedlings), inoculated stressed (water-stressed and inoculated seedlings), and non-inoculated stressed (water-stressed and non-inoculated seedlings). Transplantation of 35-day-old rice seedlings was done in earthen pots. Recommended doses of essential nutrients P and N were applied as urea (CH_4_N_2_O) and diammonium phosphate, respectively, as described by Mahreen et al. (2022). Control treatment pots were well-irrigated during the whole experiment, whereas stress-treated pots were well-irrigated at 30 days after transplantation (DAT) and then exposed to water withholding for the next 15 consecutive days. During the period of water stress, i.e., for 15 days, these pots were protected from rain and then re-irrigated till harvesting.

At the end of the water-stress imposition, three plants per replicate from each treatment pot were taken to determine the effect of bacterial inoculation on the morphological, physiological, and biochemical parameters of plants as compared with non-inoculated control. At the harvesting time, yield-related attributes of inoculated and non-inoculated stressed plants were studied.

##### 2.6.4.1 FISH-CLSM for colonization of inoculated drought-tolerant PGPB

Roots of inoculated and non-inoculated plants grown under water stress and non-stressed conditions in pots were studied at 15 days after stress (DAS) with fluorescent *in situ* hybridization-confocal laser scanning microscopy (FISH-CLSM). The bacterial population was detected through a “EUB338” FLUOS-labeled probe (Amann et al., 1995) at 448 nm using an argon ion laser. Bacterial colonization on roots was visualized by using FV10-ASW 1.7 imaging software (Manz et al., 1996).

Rhizosphere soil was studied for the re-isolation of inoculated bacteria by viable count at 15 DAS (Bernal et al., 2022). Identification of re-isolated drought-tolerant PGP bacteria was done by comparing their morphology and PGP attributes (drought tolerance, IAA production, and P solubilization) to the pure colonies of inoculated bacteria as described by Yasmin et al. (2016).

##### 2.6.4.2 *In situ* handheld techniques to study the inoculated plant responses to water stress

###### 2.6.4.2.1 Infrared thermal imaging of plants

An infrared FLIR E6 camera (FLIR Systems Inc., MA, USA) was used to capture thermal images. Inoculated and non-inoculated rice plants before and after water stress imposition were studied for thermal imaging on the 5th, 10th, and 15th DAS. The FLIR (E6) camera was used with the temperature range −20 to 250°C, IR emissivity 0.1 to 0.95, resolution 19,200 pixels (160 × 120), range of spectra 7.5–13 µm,<0.06°C thermal sensitivity, and auto modes for hot/cold detection. To minimize the background temperature of the plant, a styrofoam sheet was used. The images were taken at a 1.5-m distance from plants, and the camera automatically saved the visual images simultaneously. Analysis of IR images was done using software (FLIR Systems, Inc.) as described by Kim et al. (2020). Six thermal images of the plant from each of the genotypes per replicate were taken, and the temperature was averaged.

###### 2.6.4.2.2 Infrared gas analyzer

All the genotypes inoculated and non-inoculated under water-stressed and well-irrigated conditions were tested for different photosynthetic parameters. An infrared gas analyzer (IRGA) (LCpro-SD Portable Photosynthetic System, Ltd., UK) was used to record the data of sub-stomatal conductance, photosynthetic rate, and photosynthetic absorbance rate (PAR) of the leaf (Zahra et al., 2021).

###### 2.6.4.2.3 Soil and plant analyzer development meter

A soil and plant analyzer development (SPAD) meter (SPAD 502, Japan) was used for *in situ* leaf chlorophyll content measurement. Readings of the SPAD meter were recorded from each replication of the tested genotypes prior to stress and on the 5th, 10th, and 15th DAS. To take readings from the SPAD meter, three different positions of leaf were selected from the base of the plant. Readings of three plants per replication and three leaves per plant were averaged for the final SPAD value (Yuan et al., 2016).

##### 2.6.4.3 Morpho-physiological and biochemical parameters of plants

To study the inoculation effect of drought-tolerant consortium plants, morphological parameters were measured at 15 DAS. Three plants per replicate of each treatment were randomly collected, and root/shoot length and fresh weight were calculated. After recording the fresh weight, plants were kept at 70°C for 48 h in an oven then dry weight was measured (Zia et al., 2021).

To analyze the inoculation effect of the drought-tolerant consortium, different physio-biochemical parameters of water-stressed as well as non-stressed plants were measured, and a composite leaf sample was prepared per replicate as described by Motaleb et al. (2018).

###### 2.6.4.3.1 Determination of relative water content

The youngest fully expanded leaves were removed to measure the relative water content of the leaf. The fresh weight (FW) of leaves was measured followed by turgid weight (TW) after soaking leaf segments in distilled water for 4 h. Then, leaf segments were oven dried at 70°C for 24 h and dry weight (DW) was measured. Three leaves of each treatment per replicate were used to calculate relative water content (RWC) (Ashraf et al., 2006) using the following formula:


RWC%=(Fresh Weight−Dry Weight/Turgid Weight−Dry Weight)×100

###### 2.6.4.3.2 Determination of membrane stability index

The membrane stability index (MSI) was measured according to the method described by Ahmad et al. 2022b. To measure MSI, 0.5-cm-size discs of fully expanded leaves were washed with distilled H_2_O and soaked in a distilled water-containing glass vial for 12 h at 24°C. An electrical conductivity (EC) meter (portable EC meter, Hanna HI 9811-5^®^ Instruments, USA) was used to measure the EC of the solution. Then, the sample was autoclaved at 120°C for 20 min and EC of the solution was again measured (Rehman et al., 2016). The formula to calculate the MSI was as follows:


MSI%=1−(EC1/EC2)×100

where EC1 is the EC before autoclaving and EC2 is the EC after autoclaving.

###### 2.6.4.3.3 Leaf chlorophyll contents

Chlorophyll contents (CHL a, CHL b, and CHL t) were measured from the same leaves used for SPAD readings of inoculated and non-inoculated water-stressed plants using the method described by Chen et al. (2016). A homogenized sample of the leaf (0.1 g) was ground using chilled acetone (80%) followed by incubation for 24 h at 10°C. Then, the sample was centrifuged at 13,000 rpm for 5 min. The spectrophotometer (CamSpec UV-Vis M550, UK) was used to measure the OD of the supernatant at 470-, 663-, and 645-nm wavelengths. CHL a, b, and t were calculated by using the g formula:


CHL a=12.7(OD 663)−2.59(OD 645)

CHL b=22.9(OD 645)−4.68(OD 663)

CHL t=20.2(OD 645)+8.02(OD 663)

###### 2.6.4.3.4 Proline content determination

Proline content was measured according to the method of Bates et al. (1973). The homogenized leaf sample (0.5 g) was ground in 5-sulfosalicylic acid (3%) and centrifuged for 10 min at 14,000 rpm. 2 ml supernatant, acid ninhydrin (2 ml), phosphoric acid (6 M; 2 ml), and 2 ml glacial acetic acid were added in a test tube followed by incubation at 100°C for 60 min in the water bath. After the incubation period, an ice bath was used to cool the reaction mixture for 10 min. Toluene (4 ml) was added to the cooled reaction mixture and vortexed for 20–30 s. Chromophore (pink to red color) containing the upper organic layer was collected in a separate tube, and OD was measured at 520 nm using a spectrophotometer (CamSpec UV-Vis M550, UK). Different concentrations of L-proline were used to develop a standard curve for determining the concentration of proline in the sample (Wang et al., 2015).

###### 2.6.4.3.5 Determination of antioxidants

After the plant’s morpho-physiological analysis in response to bacterial inoculation under water stress, for biochemical analysis different antioxidant enzyme activities were studied. A homogenized leaf sample of 0.1 g was used for the determination of each enzyme analysis; the details were mentioned in our previous study (Mahreen et al., 2022). Peroxidase (POD) activity was measured using guaiacol (substrate) at 470 nm on a spectrophotometer (CamSpec M350, UV visible, UK) as described by Munawar et al. (2021). For the determination of catalase (CAT) activity, substrate H_2_O_2_ was used at 240-nm wavelength (CamSpec M350, UV visible, UK) (Chance and Maehly, 1955). Superoxidase dismutase (SOD) activity was measured using substrate nitro blue tetrazolium (NBT) reduction at 560-nm wavelength (Giaanopolitis and Ries, 1977). For the determination of polyphenol oxidase (PPO) activity, L-tyrosine (substrate) was used at 280-nm wavelength as described by Nawaz et al. (2020). The activity of phenylalanine ammonia-lyase activity (PAL) was assessed by using substrate L-phenylalanine at 290 nm as mentioned by Tovar et al. (2002).

##### 2.6.4.4 Yield attributes

Plants grown from inoculated and non-inoculated seedlings of each genotype were harvested at maturity. Different plant growth- and yield-related parameters like the number of tillers, plant height, 100-grain weight, plant weight, and paddy yield were measured from harvested plants.

### 2.7 Statistical analysis

Data from all *in vitro* and pot experiments were statistically analyzed by analysis of variance (ANOVA). Least significant difference (LSD) was used to compare different treatments at 0.01% and 0.05% confidence levels for *in vitro* and *in vivo* studies using the software Statistix 10 (Analytical Software, Tallahassee, USA). For correlation in various physio-biochemical and photosynthetic parameters, yield, and IR temperature, principal component analysis (PCA) was carried out using software SPSS 21.0 (SPSS Inc., CA, USA).

## 3 Results

### 3.1 Bacterial isolation and screening for drought tolerance

Two hundred and fifty-five different morphotypes were isolated and screened for drought tolerance using PEG-8000-supplemented LB medium. Out of 255 bacteria, 25 were found to be drought tolerant as indicated by viability from PEG-containing medium and expressed as drought tolerance ranging from 15% to 20% (**Figure S1**). Among 25 drought-tolerant bacteria, five bacteria were isolated from Sheikhupura, six from Nankana Sahib, five from Gujranwala, four from Pindi Bhattian, three from Faisalabad, and two from Kala Shah Kaku (**Figure S1**).

### 3.2 Bioassay for PGP traits and identification of drought-tolerant bacteria

Out of 25 drought-tolerant bacteria, eight bacterial strains (NM-1, NM-2, NM-3, NM-4, NM-5, NM-6, NM-7, and NM-8) showed *in vitro* drought-stress tolerance (20%) and plant growth-promoting activities (**Table S1, 2**). The morphological characteristics of eight selected bacteria were studied using a light microscope (**Table S1**). These bacteria belonged to genera *Bacillus*, *Brucella*, and *Ochrobactrum* based on 16S rRNA gene sequencing (accession numbers OP363606, OP363607, OP363608, OP363609, OP363610, OP363611, OP363612, and OP363613) (**Table S1**). The maximum likelihood method was used to construct a phylogenetic tree using Molecular Evolutionary Genetics Analysis Version 11 (MEGA 11) software (**Figure S2**). The phosphate solubilization index (SI) for these bacteria was observed on NBRIP medium ranging from 1.9 to 5.6, whereas the change in pH ranged from 3.67 to 6.55 (**Table 1**, **Figure S3**). A maximum P solubilization index (SI: 5.6) was observed for *Brucella* sp. NM-4 followed by *Bacillus cereus* NM-6 (SI: 5.2) (**Table 1**). The maximum change in pH (3.67) was observed for *Brucella* sp. NM-4 (**Figure S3**). No halo zone was observed by the selected drought-tolerant bacteria on blood agar plates.

**Table 1:** Plant growth-promoting traits and organic acids produced by drought-tolerant bacteria.

Drought tolerant bacteria	*Ochrobactrum soli* NM-1	*Bacillus subtilis* NM-2	*Brucella anthropi* NM-3	*Brucella haematophilum* NM-4	*Bacillus* sp. NM-5	*Bacillus cereus* NM-6	*Bacillus clarus* NM-7	*Bacillus australimaris* NM-8
Phosphate solubilization								
**Phosphate solubilization (SI)^a^**	2.8 ± 0.1	5 ± 0.2	2.3 ± 0.1	5.6 ± 0.2	1.9 ± 0.1	5.2 ± 0.2	4.9 ± 0.2	1.9 ± 0.1
**Solubilized P (µg mL^-1^)^b^**	140 ± 3.5b	313 ± 7.3a	82 ± 3bc	356 ± 7.5a	52 ± 3c	315 ± 5.2a	302 ± 4.6a	51 ± 3c
Organic acids produced (µg mL^-1^)^c^								
**Citric acid**	12.2 ± 0.3d	25.1 ± 1.2b	13.2 ± 0.3d	71.0 ± 3.0a	11.2 ± 0.8d	12.5 ± 0.5d	18.3 ± 0.8c	10.4 ± 0.5d
**Acetic acid**	70 ± 3.0d	153 ± 4.7bc	36 ± 1.5e	178 ± 8.0a	163 ± 6.7b	151 ± 4.0bc	161 ± 6.0b	144 ± 5.5c
**Gluconic acid**	63 ± 2.0d	134 ± 7.5a	33 ± 1.0e	123 ± 5.5b	23 ± 1.0e	118 ± 5.5bc	110 ± 4.5c	32 ± 1.5e
**Malic acid**	24 ± 1.5d	73 ± 3.0b	20 ± 0.6d	86 ± 4.0a	5.7 ± 0.2f	39 ± 2.0c	15 ± 0.5e	14 ± 0.3e
**Gibberellic acid**	13 ± 0.5d	20 ± 1.0b	19 ± 0.6b	30 ± 1.5a	5.2 ± 0.2f	12 ± 0.5d	16 ± 0.5c	9 ± 0.2e
**Oxalic acid**	8 ± 0.3e	12 ± 0.5d	5 ± 0.2f	20 ± 1.0a	7 ± 0.3e	18 ± 0.7b	16 ± 0.6c	8 ± 0.2e
**Succinic acid**	20 ± 1.0cd	72 ± 3.0b	23 ± 1.0c	82 ± 4.0a	16 ± 0.5de	78 ± 3.0a	13 ± 0.5e	7 ± 0.2f
Plant growth-promoting traits								
**IAA production (µg mL^-1^)^d^**	22.2 ± 0.8	31.5 ± 1.5	23.0 ± 1.0	46.2 ± 1.3	15.7 ± 0.6	37.5 ± 1.3	30.2 ± 0.8	14.3 ± 0.6
**ACC deaminase activity ^e^**	–	++	–	++	++	++	+	++
**Siderophore production ^f^**	+	++++	++	++++	++	++++	+++	++
**EPS production (R_f_) ^g^**	–	0.5	–	0.4	0.3	0.3	–	–

**^a^** P solubilization index (PSI) was measured from halo zone formation on a plate containing NBRIP agar having tricalcium phosphate (TCP) as insoluble P source.

**^b^** Solubilized P by drought-tolerant bacteria was quantified using a spectrophotometer in liquid NBRIP medium.

**^c^** Production of organic acids by drought-tolerant bacteria in NBRIP broth at the 7th day during in vitro phosphate solubilization was analyzed on HPLC.

**^d^** Indole-acetic acid (IAA) quantification was done by HPLC.

**^e^** ACC (1-aminocyclopropane-1-carboxylic acid) deaminase was analyzed qualitatively by using DF minimal medium supplemented with ACC as a sole source of nitrogen; + shows growth, and − shows no growth.

**^f^** Production of siderophores by tested bacteria was studied qualitatively using CAS medium. + shows level of production, and – shows no production of siderophores.

**^g^** Production of exopolysaccharides (EPS) was studied quantitatively through thin-layer chromatographic (TLC) assay. R_f_ = retention factor. symbol +, ++, +++ and ++++ showed qualitatively level of production of ACC deaminase and siderophores. "+" showed minimum level while "++++" showed maximum level of production.

**Figure 1:**
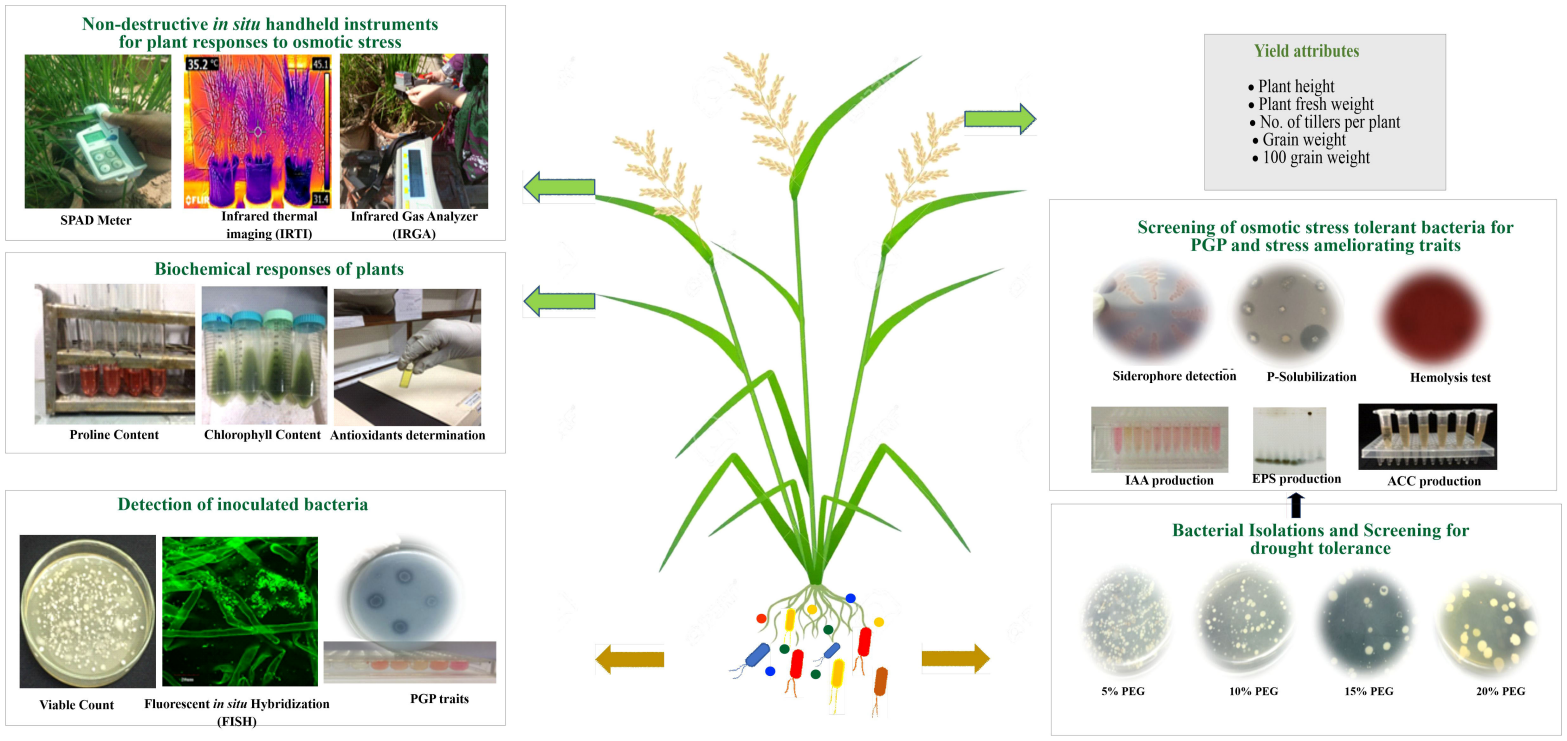
Illustrations of workflow for isolation, molecular characterization of PGPB, and experimental setup for plant responses to inoculation through biochemical and handheld techniques.

These potential drought-tolerant bacteria produced IAA ranging from 14.3 to 46.2 µg ml^-1^. Higher IAA (46.2 µg ml^-1^) was produced by *Brucella* sp. NM-4 followed by *Bacillus cereus* NM-6 (37.5 µg ml^-1^) and *Bacillus subtilis* NM-2 (31.5 µg ml^-1^) (**Table 1**). *Bacillus subtilis* NM-2, *Brucella haematophila* NM-4, *Bacillus cereus* NM-6, and *Bacillus clarus* NM-7 showed ACC deaminase activity and siderophore production (**Table 1**). *Bacillus subtilis* NM-2 showed production of fructose (polysaccharide), as indicated by the highest retention factor (R_f_) of 0.5 on TLC (**Figure 1**).

### 3.3 Production of organic acids

Drought-tolerant PGP bacteria were found to produce multiple organic acids, i.e., citric acid (10.4–71.0 µg ml^-1^), acetic acid (36–178 µg ml^-1^), gluconic acid (23–134 µg ml^-1^), malic acid (5.7–86 µg ml^-1^), and succinic acid (7–82 µg ml^-1^). Gibberellic acid (5.2–30 µg ml^-1^) was produced by *Bacillus subtilis* NM-2, *Brucella anthropi* NM-3, *Brucella haematophila* NM-4, and *Bacillus* sp. NM-5, whereas oxalic acid (5–20 µg ml^-1^) was produced by *Brucella anthropi* NM-3, *Brucella haematophila* NM-4, *Bacillus cereus* NM-6, and *Bacillus clarus* NM-7 (**Table 1**).

### 3.4 Effect of drought-tolerant bacteria on rice seedling’s vigor and root morphology

Seed germination assay showed a positive effect of the tested bacteria on seed germination and seedlings of three rice genotypes under 20% PEG osmotic stress as compared with the non-inoculated stressed seedlings. The maximum vigor index (1009.2-1100) was observed for three tested genotypes in response to inoculation with consortium followed by *Brucella haematophila* NM-4 (957-1046.7) with PEG-8000-induced stress (**Table S2**).

**Figure 2:**
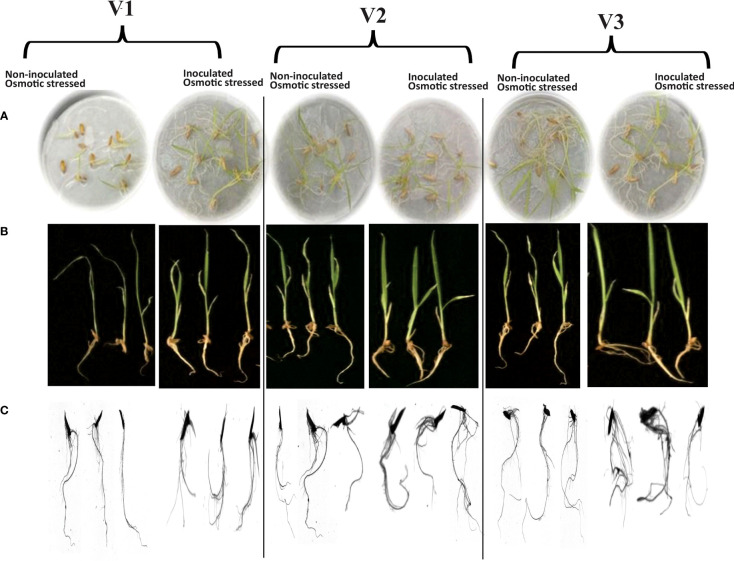
Effect of drought-tolerant consortium on the growth of rice genotypes in plate assay under 20% PEG-8000 mediated stress **(A)**. Root/shoot lengths of the seedlings to measure the vigor index **(B)**. Rhizo-scanning shows the root morphology **(C)** of non-inoculated and consortium-inoculated roots under osmotic stress. Data were recorded at the 7th DPI of seed germination in a plate assay. Three biological replicates per treatment were analyzed for different root parameters using the Rhizo scanner.

Rhizoscanning of rice genotypes showed improved root length (17%) in V3 followed by a 10% increase in V2 as compared with V1 in response to inoculation with the consortium (*Bacillus subtilis* NM-2, *Brucella haematophila* NM-4, and *Bacillus cereus* NM-6) under osmotic stress (**Figure 2**; **Table 2**). Inoculation also improved other parameters, i.e., length per volume (19%) increased in V3 followed by (12%) in V2 as compared with V1 and average diameter (18%) increased in V3 as compared with V1 under 20% PEG stress (**Table 2**).

**Table 2:** Rhizo-scanning to study the effect of the drought-tolerant bacterial consortium on root parameters of rice seedlings grown at 20% PEG-mediated osmotic stress under growth room conditions.

Treatments	Genotypes	Root length (cm)	Projection area (cm^2^)	Surface area (cm^2^)	Average diameter (mm)	Length per volume (cm/m^3^)	Root volume (cm^3^)
**Control**	**V1**	27.33 ± 1.29ab	0.97 ± 0.04ab	3.04 ± 0.13ab	0.35 ± 0.01ab	27.33 ± 1.36abc	0.028 ± 0.002a
	**V2**	26.55 ± 1.30ab	0.85 ± 0.03ab	2.65 ± 0.10ab	0.32 ± 0.02ab	26.55 ± 1.32abc	0.022 ± 0.002a
	**V3**	27.37 ± 1.32ab	0.93 ± 0.03ab	2.91 ± 0.13ab	0.34 ± 0.03ab	27.37 ± 1.36bc	0.025 ± 0.002a
**Inoculated control**	**V1**	28.51 ± 1.41ab	1.02 ± 0.06ab	3.21 ± 0.16ab	0.37 ± 0.02ab	29.51 ± 1.47bc	0.028 ± 0.002a
	**V2**	29.51 ± 1.39ab	1.02 ± 0.04ab	3.21 ± 0.16ab	0.35 ± 0.03ab	29.51 ± 1.43bc	0.028 ± 0.001a
	**V3**	29.58 ± 1.45ab	0.92 ± 0.05ab	2.88 ± 0.15ab	0.31 ± 0.01ab	29.58 ± 1.45bc	0.023 ± 0.001a
**Osmotic stressed**	**V1**	16.35 ± 0.80b	0.66 ± 0.03b	2.09 ± 0.10b	0.19 ± 0.01b	19.07 ± 0.95c	0.018 ± 0.001a
	**V2**	20.04 ± 1.10ab	0.81 ± 0.02ab	2.56 ± 0.13ab	0.27 ± 0.01b	20.04 ± 1.02c	0.020 ± 0.001a
	**V3**	22.56 ± 1.13ab	0.84 ± 0.04ab	2.63 ± 0.16ab	0.30 ± 0.01b	22.56 ± 1.12bc	0.025 ± 0.002a
**Inoculated osmotic stressed**	**V1**	30.25 ± 1.50ab	0.92 ± 0.05ab	2.88 ± 0.14ab	0.31 ± 0.01ab	29.58 ± 1.45bc	0.023 ± 0.003a
	**V2**	33.72 ± 1.54a	0.91 ± 0.05ab	2.87 ± 0.14ab	0.31 ± 0.05a	33.72 ± 1.68b	0.026 ± 0.003a
	**V3**	36.52 ± 1.67a	1.10 ± 0.07a	3.46 ± 0.20a	0.38 ± 0.04ab	36.52 ± 1.52a	0.026 ± 0.002a

Effect of the drought-tolerant bacterial consortium on root parameters of rice genotypes; V1: Super Basmati, V2: NIBGE-DT02, and V3: IR55419-04. Seeds were soaked in the consortium (Bacillus subtilis NM-2, Brucella haematophilum NM-4, and Bacillus cereus NM-6: 1 × 10^8^ CFU ml^-1^) for 30 min prior to being kept on filter paper in a petri plate. Control seeds were dipped in non-inoculated LB broth. Treatments; Control (non-inoculated seeds + Hoagland solution), inoculated control (inoculated seeds + Hoagland solution), osmotic stressed (non-inoculated seeds + 20% PEG-8000-supplemented Hoagland solution), and inoculated osmotic stressed (inoculated seeds + 20% PEG-8000-supplemented Hoagland solution). Plates were incubated in growth room at 28 ± 2^°^C for 7 days. Data were recorded at 7th DPI, and all values are an average of three biological replicates per treatment. ± shows the standard deviation. Non-significant means are followed by same letter at p = 0.01 according to LSD. Different letters a, b and c showed significant data while similar letters showed non-significance.

### 3.5 *In planta* evaluation of drought-tolerant bacteria on rice under water stress in a pot experiment

#### 3.5.1 *In situ* handheld techniques to study the inoculated plant responses to water stress

##### 3.5.1.1 Infrared thermal imaging of inoculated plants under water stress

**Figure 3:**
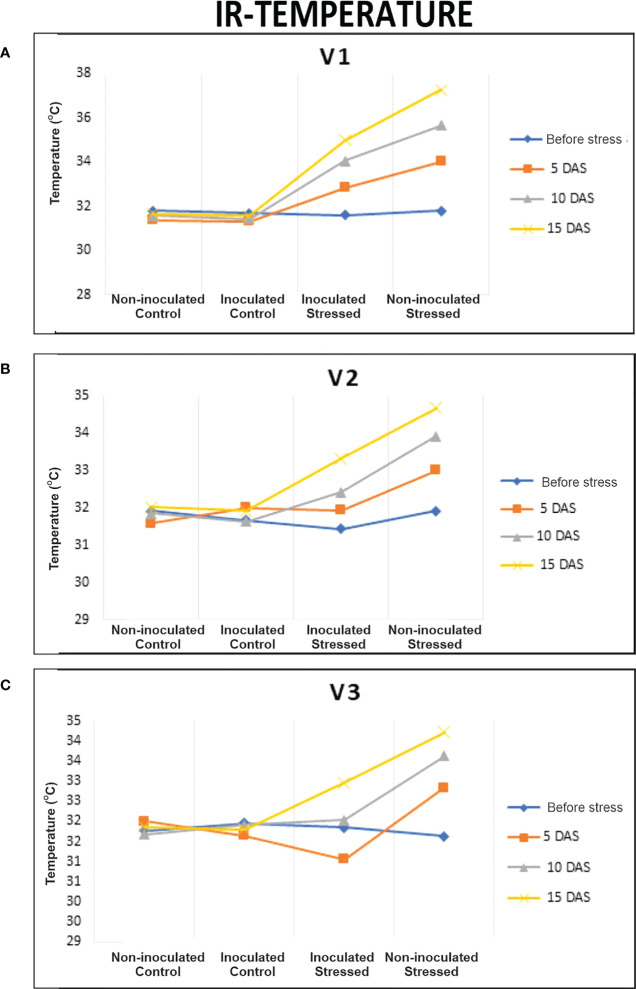
Evaluation of the drought-tolerant PGPB consortium on plant temperature using infrared thermal imaging (IRTI) in pot experiment under net house conditions. Rice genotypes; V1: Super Basmati, V2: NIBGE-DT02 and V3: IR55419-04. **(A)** IR temperature (°C) of V1 plants, **(B)** IR temperature (°C) of V2 plants, and **(C)** IR temperature (°C) of V3 plants in different treatments; non-inoculated control, inoculated control, inoculated stressed, and non-inoculated stressed. Data were an average of six biological replicates.

**Figure 4:**
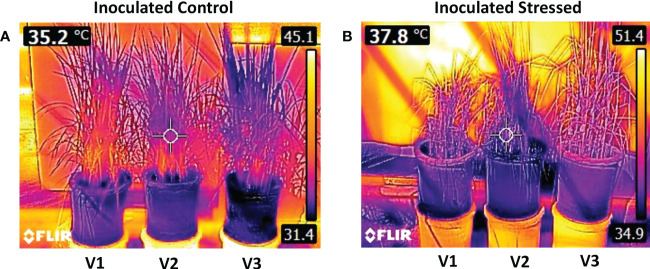
Infrared thermal images of rice genotypes taken by a FLIR T-E6 camera in a pot experiment under the net house. Rice genotypes V1: Super Basmati (SB), V2: NIBGE-DT02, and V3: IR55419-04. **(A)** Thermal image of control-inoculated plants taken at 15 days after stress (DAS) and **(B)** thermal image of inoculated stressed plants taken at 15 DAS.

During 15 days of water withholding, plant temperatures of all genotypes at 0 days after stress (DAS) ranged between 31.4 and 32°C. The temperature difference between non-inoculated and inoculated stressed plants was found to be significant at 15 DAS (**Figure 3**). A maximum increase in plant temperature was observed for non-inoculated stressed V1 (17.7%) as compared with non-inoculated well-irrigated control (**Figure 3**). Inoculated stressed plants of all genotypes showed less increase in temperature (33–35°C) as compared with the non-inoculated stressed plants (34.2–37.3°C) (**Table S3**). A percent decrease in temperature at 15 DAS was observed for inoculated V3, i.e., 3.6% followed by V2 (4.2%) as compared with non-inoculated stressed (**Figures 3**, **4**; **Table S3**).

##### 3.5.1.2 Infrared gas analyzer for photosynthetic characteristics

Plant parameters related to photosynthesis, i.e., reference CO_2_ (cref), photosynthetically active radiation (PAR) at leaf surface (Qleaf), sub-stomatal CO_2_ (ci), transpiration rate (E), and photosynthetic rate (A), were reduced with the increased water stress in all tested genotypes (**Table 3**). Consortium inoculation significantly improved the cref (395–411 ppm) in tested genotypes under stressed plants (**Table 3**). Relatively least reduction in PAR (1.4%) was recorded in inoculated V3 followed by 2.3% in V2 under stressed conditions. Consortium inoculation showed a higher value (1,509 µmol s^-1^ m^-2^) of PAR in V2 plants under stressed conditions as compared with non-inoculated stressed plants (695 µmol s^-1^ m^-2^). Consortium inoculation showed a higher value (788 µmol mol^-1^) of ci in the V3-stressed plants as compared with the non-inoculated stressed plants (428 µmol mol^-1^) (**Table 3**).

**Table 3:** Effect of inoculation on rice plants’ photosynthetic parameter using infrared gas analyzer (IRGA) in a pot experiment under water stress conditions.

Treatments	Genotypes	Photosynthetic parameters				
		Reference CO_2_ (cref)^a^	PAR at leaf surface (Qleaf)^b^	Sub-stomatal CO_2_ (ci)^c^	Transpiration rate (E)^d^	Photosynthetic rate (A)^e^
**Non-inoculated control**	V1	406.3 ± 2.5cd	795 ± 17.9c	424.3 ± 12.6c	235.82 ± 13.9e	25.8 ± 1.7d
	V2	399 ± 1.7cd	711.7 ± 20.1d	413.3 ± 20.2d	398.98 ± 15.4bc	24.27 ± 0.9d
	V3	409 ± 3cd	804.3 ± 25.9c	446.3 ± 3.5c	393.31 ± 11.5c	25.13 ± 2.1d
**Inoculated control**	V1	485 ± 13.5a	1705.3 ± 32.9a	856.3 ± 41.3a	473.67 ± 25.8a	66.97 ± 2.8a
	V2	492 ± 8.5a	1655 ± 36.7a	801.7 ± 27.5b	420.24 ± 9.3b	60.3 ± 2.5ab
	V3	440.7 ± 9.5b	1710.3 ± 29a	845 ± 21.7a	473.67 ± 24.2a	66.97 ± 7.2a
**Inoculated stressed**	V1	411 ± 4.6c	1490.7 ± 31.7d	786 ± 11.3d	182.73 ± 9.9f	18.63 ± 0.8d
	V2	395 ± 4de	1509 ± 44.9b	781.3 ± 10b	302.49 ± 15.2d	51.97 ± 11.8bc
	V3	400 ± 10.4de	1515.7 ± 20.07b	788 ± 21.5b	314.51 ± 19.4d	50.63 ± 9.6c
**Non-inoculated stressed**	V1	393.3 ± 10.1de	572 ± 22.9e	329 ± 20.1e	167.61 ± 10.8f	17.6 ± 1d
	V2	382.3 ± 9.3e	695.3 ± 12.3d	328.3 ± 18.9e	232.82 ± 8.6e	21.97 ± 2.9d
	V3	395 ± 9.9de	793.3 ± 15.6c	427.7 ± 17.6c	245.49 ± 13.6e	22.8 ± 1.9d

Evaluation of inoculation under water stress on plant photosynthesis related parameters:

^a^Reference CO_2_ (ppm),

^b^PAR at leaf surface (µmol s^-1^ m^-2^),

^c^sub-stomatal CO_2_ (µmol mol^-1^),

^d^transpiration rate (mol m^-2^ s^-1^), and

^e^photosynthetic rate (µmol m^-2^ s^-1^) of rice genotypes; V1: Super Basmati, V2: NIBGE-DT02, and V3: IR55419-04 under net house conditions. Treatments: non-inoculated control, inoculated control, inoculated stressed, and non-inoculated stressed. Mean data were represented and means are an average of six-biological replicates, and each replicate has three plants (10 leaves per plant) in the homogenized sample. Means with the same letter showed non-significant data at p = 0.05, and those with different letters differ significantly according to LSD.

In all the tested genotypes, inoculation improved E of plants ranging from 182.7 to 314 mol m^-2^ s^-1^ under stress as compared with the non-inoculated stressed plants (**Table 3**). Inoculation improved A (60.3–66.9 µmol m^-2^ s^-1^) in well-irrigated plants and in stressed plants (18.6–51.9 µmol m^-2^ s^-1^) (**Table 3**).

##### 3.5.1.3 Soil and plant analyzer development for chlorophyll content

**Figure 5:**
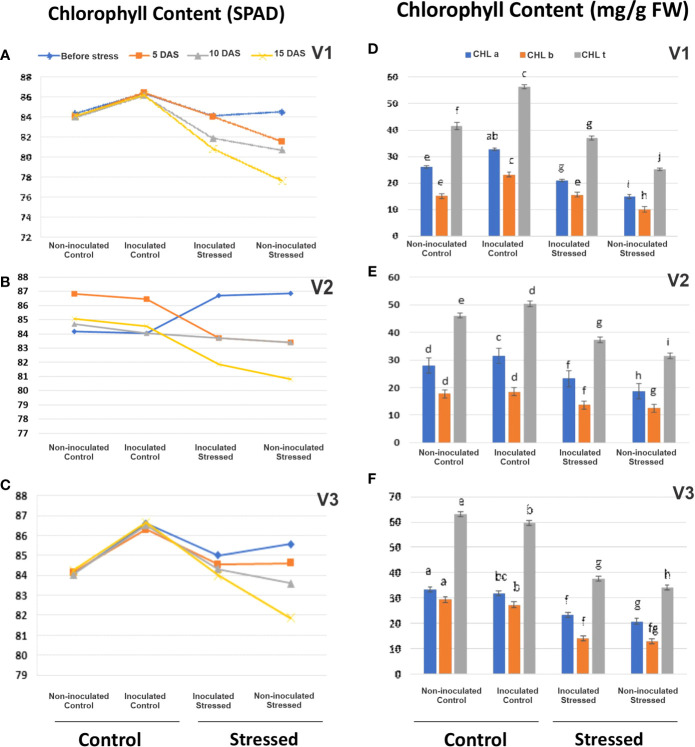
Evaluation of the drought-tolerant consortium on plant chlorophyll content in a pot experiment under net house conditions. Using the soil plant analyzer development (SPAD) meter **(A–C)** and biochemical analysis of chlorophyll a, b, and t (mg/g FW) **(D–F)** in rice genotypes, V1: Super Basmati (SB), V2: NIBGE-DT02, and V3: IR55419-04 under different treatments. Non-inoculated control, inoculated control, inoculated stressed, and non-inoculated stressed. Data were averaged for six biological replicates. Mean data were represented, and different letters showed that that data were significant at p = 0.05 according to LSD.

Plant chlorophyll content decreased variably in all the tested genotypes with the increasing water stress as indicated by SPAD meter values (**Figures 5A–C**; **Table S4**). SPAD showed improvement in V2-inoculated plants (3%) at 15 DAS as compared with its respective non-inoculated stressed plants (**Figure 5A**; **Table S4**).

#### 3.5.2 Morpho-physiological and biochemical parameters of inoculated plants

**Figure 6:**
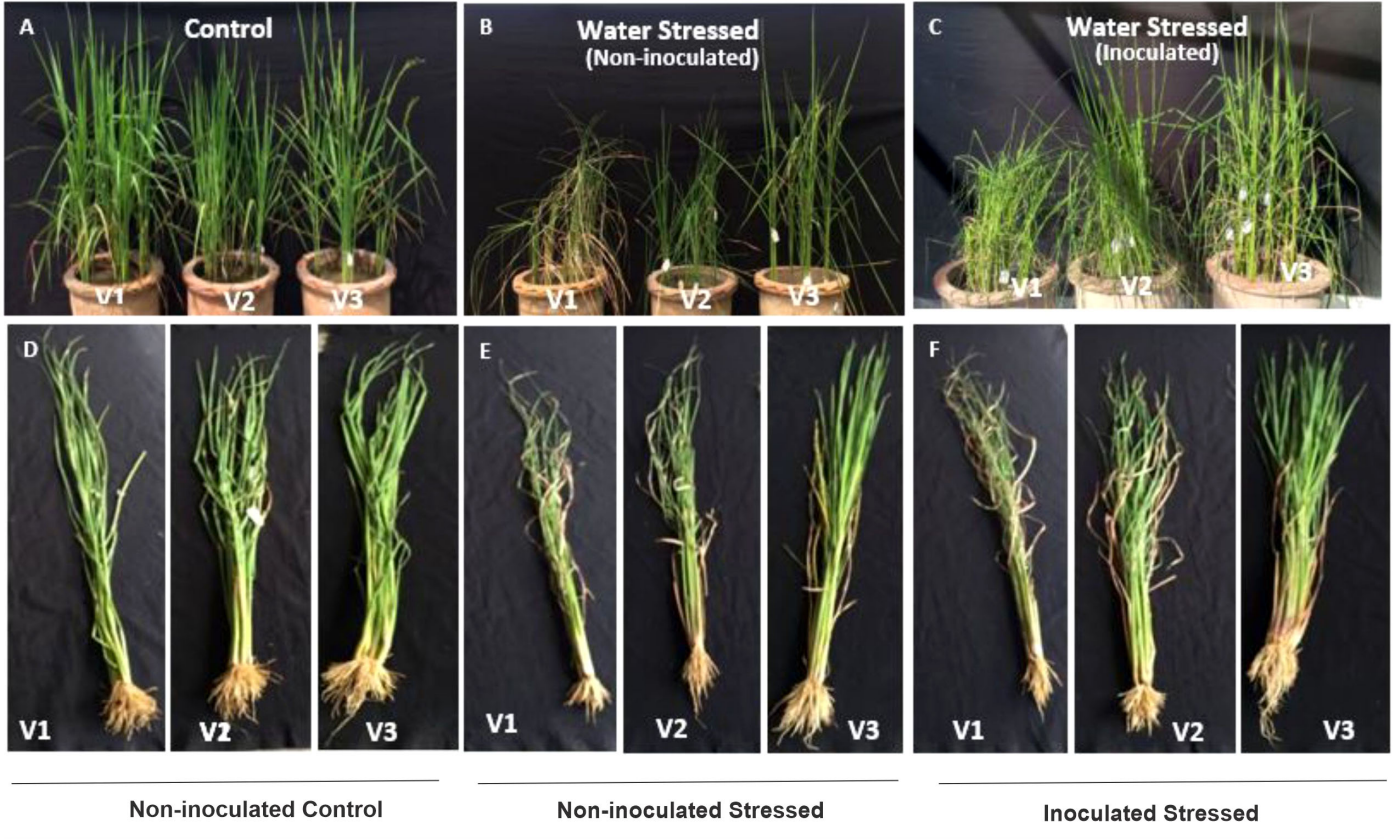
Morphological representation of plants grown in pots under net house conditions. Rice genotypes; V1: Super Basmati (SB), V2: NIBGE-DT02, and V3: IR55419-04. **(A)** Control-well-irrigated rice plants. **(B)** Water-stressed non-inoculated plants. **(C)** Water-stressed inoculated plants. **(D–F)** Shoot and root lengths of rice plants of three genotypes under different treatments of control and water stress.

**Figure 7:**
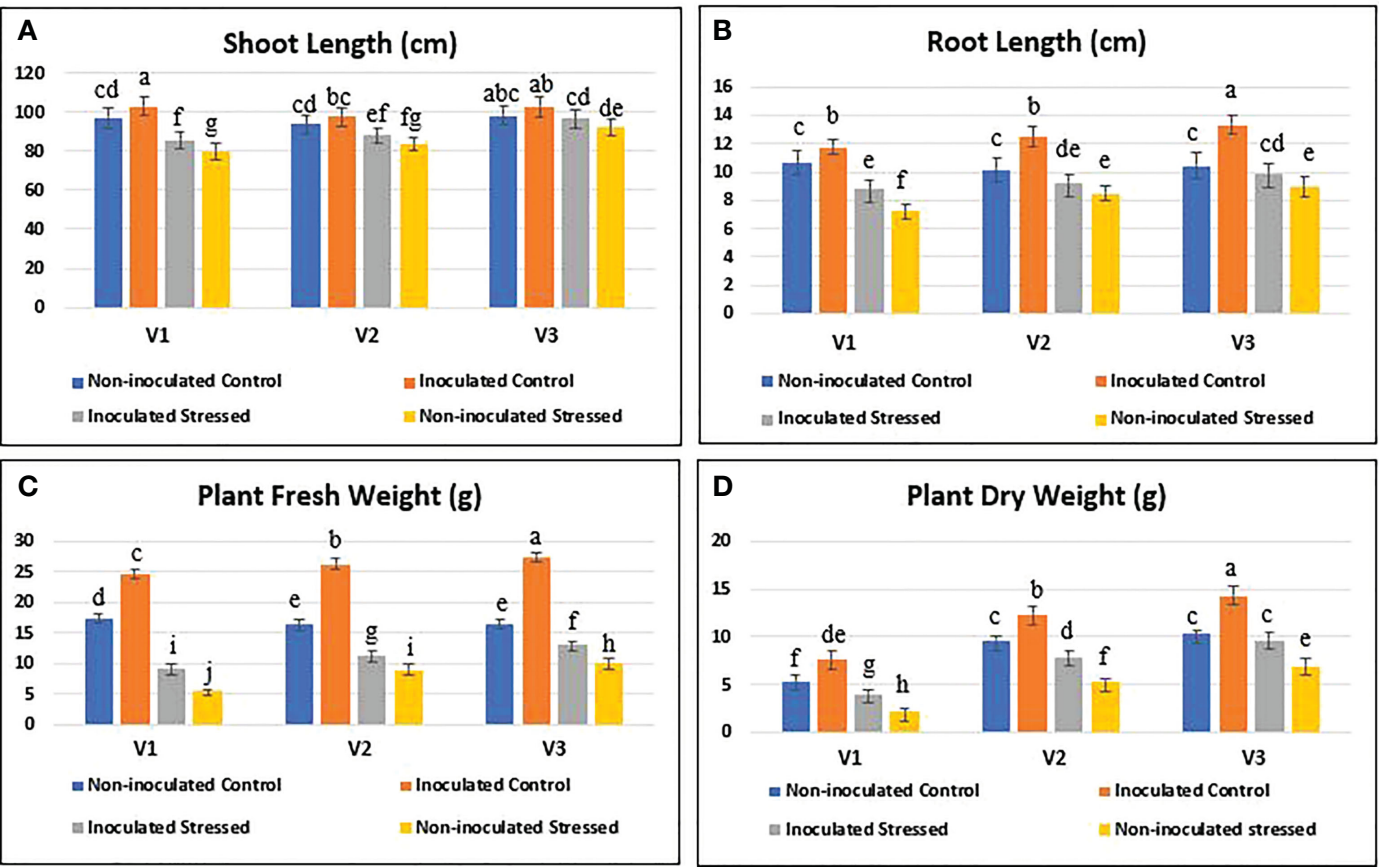
Effect of the drought-tolerant PGPB consortium on plant growth parameters in a water stress pot experiment under net house conditions. Rice genotypes V1: Super Basmati (SB), V2: NIBGE-DT02, and V3: IR-55419. **(A)** Shoot length (cm). **(B)** Root length (cm). **(C, D)** Plant fresh and dry weights (g) under treatments; non-inoculated control, inoculated control, inoculated stressed, and non-inoculated stressed. Data were an average of six biological replicates. Mean data were represented and means with the same letter differ non-significantly, and different letters showed that the data were significant at p = 0.05 according to LSD.

Plant growth in all tested rice genotypes was decreased under water-deficit conditions as compared with their respective well-irrigated controls (**Figure 6**). Root/shoot lengths of three genotypes during 15 days of water withholding were significantly increased with the drought-tolerant bacterial consortium as compared with the non-inoculated stressed plants (**Figures 7A, B**). The plant fresh weight of inoculated plants increased (9–13 g) as compared with non-inoculated stressed plants (5.7–10 g) (**Figure 7C**). Inoculation also improved the plant’s dry weight (38.7–85.7%) as compared with non-inoculated water-stressed plants (**Figure 7D**).

##### 3.5.2.1 Chlorophyll content of plants

Consortium inoculation stimulated the accumulation of total chlorophyll content ranging 36.97–37.63 mg g^-1^ f. wt. in stressed leaves as compared with non-inoculated stressed leaves (25.27–33.9 mg g^-1^ f. wt.) (**Figures 5D–F**; **Table S5**). Accumulation of CHL b (37.25 mg g^-1^ f. wt.) was observed in inoculated V2 leaves under stress as compared with non-inoculated stressed leaves, i.e., 31.57 mg g^-1^ f. wt. (**Figure 5D**; **Table S5**).

##### 3.5.2.2 Relative water content, membrane stability index, and proline content of plants

Inoculation of plants with the drought-tolerant consortium showed improved RWC as compared with the non-inoculated plants (**Table 4**). RWC for inoculated stressed plants of all genotypes ranged between 47 and 51% as compared with non-inoculated stressed plants (32–43%) (**Table 4**). Maximum stimulation (51%) of RWC was observed in inoculated V3 leaves followed by V2 (50%) and V1 (47%) (**Table 4**).

**Table 4:** Effect of inoculation on rice plant relative water content, membrane stability index, proline content, and antioxidant enzymes in a pot experiment under water stress conditions.

Treatments	Genotypes	RWC%^a^	MSI%^b^	Proline content^i^	Antioxidant enzymes^c^				
					POD^f^	CAT^e^	PPO^g^	SOD^h^	PAL^d^
**Non-inoculated control**	V1	53.33 ± 2.53a	68.58 ± 3.21a	39.36 ± 1.73c	371.00 ± 14.9a	1.02 ± 0.043cd	14.00 ± 0.5cd	1.84 ± 0.06def	0.74 ± 0.02c
	V2	53.33 ± 2.41a	68.60 ± 3.23a	39.54 ± 1.65c	373.83 ± 16.5a	0.98 ± 0.042cd	18.76 ± 0.7bc	1.80 ± 0.07def	0.74 ± 0.03c
	V3	53.70 ± 2.15a	68.82 ± 3.15a	35.58 ± 1.52c	376.67 ± 16.8a	1.02 ± 0.048cd	15.83 ± 0.5cd	1.91 ± 0.08def	0.75 ± 0.03c
**Inoculated control**	V1	54.17 ± 2.62a	69.66 ± 3.42a	38.96 ± 1.77c	370.33 ± 15.1a	1.02 ± 0.039cd	13.23 ± 0.4d	1.89 ± 0.05def	0.73 ± 0.01c
	V2	52.38 ± 2.45a	69.72 ± 3.45a	39.23 ± 1.82c	374.33 ± 16.8a	1.00 ± .037cd	19.03 ± 0.8bc	1.68 ± 0.06ef	0.71 ± 0.02c
	V3	53.98 ± 2.58a	69.46 ± 3.44a	36.57 ± 1.47c	375.33 ± 15.3a	1.00 ± 0.034cd	15.33 ± 0.6cd	1.84 ± 0.06def	0.74 ± 0.02c
**Inoculated stressed**	V1	46.67 ± 1.85ab	59.49 ± 2.33ab	118.15 ± 3.39ab	315.33 ± 14.2ab	1.50 ± 0.063abc	21.53 ± 0.9ab	2.42 ± 0.08cd	0.93 ± 0.04bc
	V2	50.16 ± 2.25a	61.73 ± 2.75ab	168.89 ± 7.23a	402.67 ± 17.2a	1.66 ± 0.054ab	24.59 ± 1.1a	3.11 ± 0.10ab	1.18 ± 0.05ab
	V3	51.37 ± 2.17a	63.85 ± 2.79ab	147.42 ± 6.23a	407.67 ± 18.1a	1.80 ± 0.063a	24.03 ± 0.9ab	3.52 ± 0.11a	1.32 ± 0.06a
**Non-inoculated stressed**	V1	32.02 ± 1.31b	49.73 ± 2.24b	54.97 ± 2.43c	175.77 ± 6.5b	0.80 ± 0.031d	11.98 ± 0.3d	1.33 ± 0.04f	0.62 ± 0.03c
	V2	39.68 ± 1.78ab	58.61 ± 2.34ab	75.92 ± 2.73bc	388.00 ± 15.1a	1.22 ± 0.019bcd	22.88 ± 0.9ab	2.26 ± 0.08cde	0.94 ± 0.04bc
	V3	42.59 ± 1.85ab	60.95 ± 2.41ab	79.31 ± 2.83bc	389.33 ± 14.7a	1.40 ± 0.019abc	18.74 ± 0.8bc	2.62 ± 0.09bc	0.90 ± 0.04bc

Evaluation of consortium inoculation under water stress on

**^a^** RWC—relative water content,

**^b^** MSI—membrane stability index,

**^c^—**antioxidant enzymes;

**^d^** PAL activity (phenylalanine ammonia lyase) = µm cinnamic acid g^-1^. f. wt.,

**^e^** CAT activity (catalase) = units g^-1^. f. wt.,

**^f^** POD activity (peroxidase) = units g^-1^. f. wt.,

**^g^** PPO activity (polyphenol oxidase) = units g^-1^. f. wt.

**^h^** SOD activity (superoxide dismutase) = units mL^-1^, and

**^i^** proline content (µM/g. f. wt.) of different rice genotypes; V1: Super Basmati, V2: NIBGE-DT02, and V3: IR55419-04 under net house conditions. Treatments: non-inoculated control, inoculated control, inoculated stressed, and non-inoculated stressed. Mean data were represented and means are an average of six-biological replicates, and each replicate has three plants (10 leaves per plant) in the homogenized sample. Means with the same letter showed non-significant data at p = 0.05 and with different letters differ significantly according to LSD.

MSI for inoculated stressed plants of all genotypes ranged between 59 and 63% as compared with non-inoculated stressed plants (50–61%) (**Table 4**). Maximum stimulation (64%) of the MSI was observed in inoculated V3 leaves followed by V2 (62%) and V1 (59%) (**Table 4**).

Stress osmolyte and proline content was higher in all the genotypes with increasing water scarcity ranging from 54.97 to 79.31 µmol g^-1^. f. wt. as compared with well-irrigated controls (35.58–39.54 µmol g^-1^. f. wt.) (**Table 4**). Inoculation of the drought-tolerant consortium stimulated the stressed plants to accumulate 169 µmol g^-1^. f. wt., increasing proline content in V2 followed by 147 µmol g^-1^. f. wt. in V3 (**Table 4**).

##### 3.5.2.3 Antioxidant enzymes

Drought stress modulated different plant enzymatic antioxidants (CAT, SOD, POD, PAL, and PPO) in all the tested rice genotypes except in V1 as compared with well-irrigated controls (**Table 4**). Consortium-inoculated plants of V2 and V3 showed that 7.7 and 8.2% increased accumulation of POD, respectively, as compared with the control plants (**Table 4**), whereas POD activity was increased to 3.7% in consortium-inoculated V2-stressed plants as compared with non-inoculated stressed plants (**Table 4**). Consortium inoculation improved the production of CAT activity by 69.4 and 76.5% in V2 and V3, respectively, under stress as compared with well-irrigated control (**Table 4**). SOD activity was increased (72.8% and 84.3%) in consortium-inoculated stressed rice genotypes V2 and V3, respectively, as compared with the well-irrigated controls (**Table 4**). Inoculation significantly increased (8%) the PPO activity in V2 plants as compared with the non-inoculated stressed plants. Consortium-inoculated V2 showed 27% increased PAL activity followed by V1 (25.7%) as compared with the control plants (**Table 4**).

### 3.6 Detection of inoculated drought-tolerant bacteria

**Figure 8:**
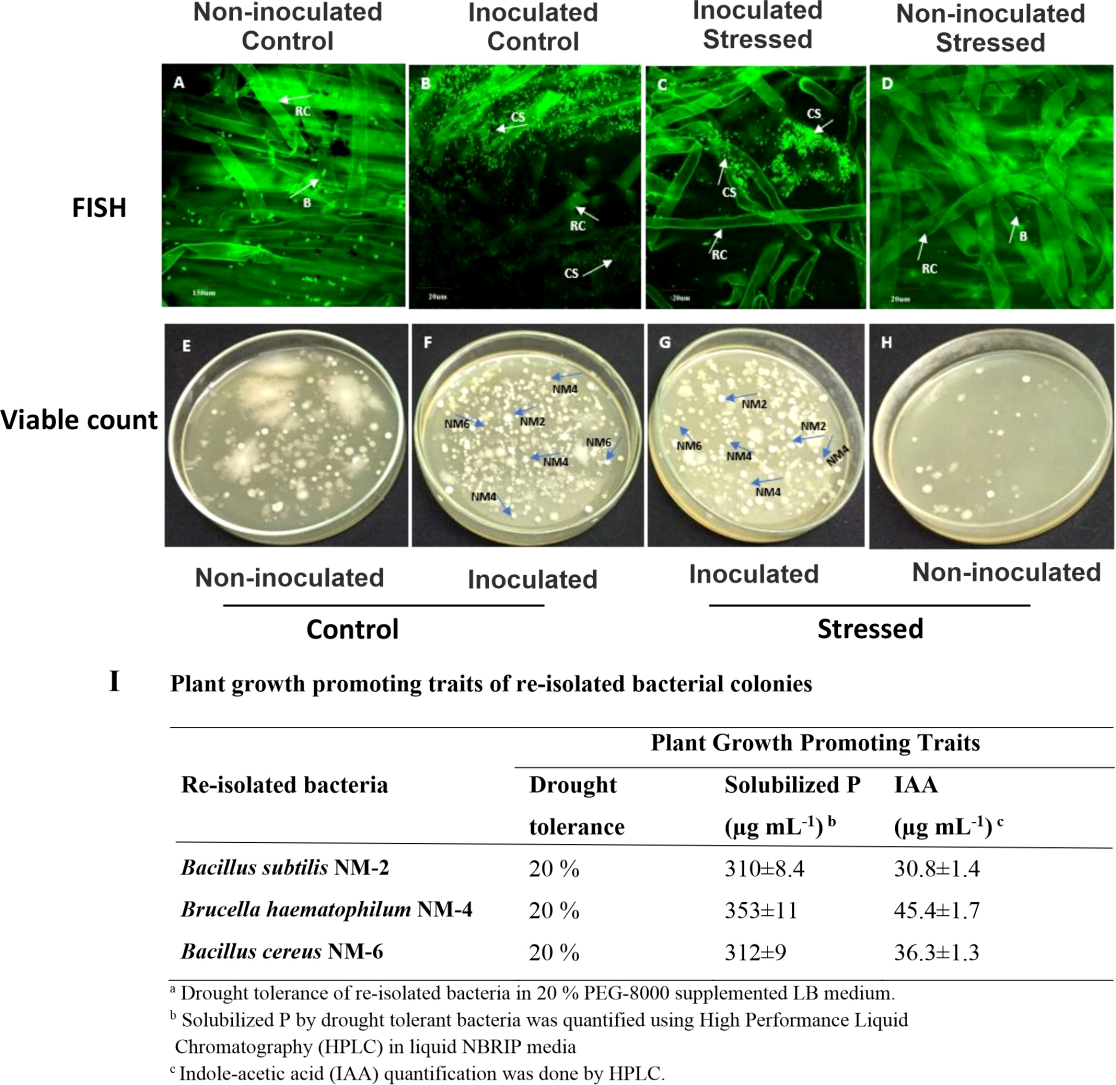
Detection of inoculated drought-tolerant bacteria. The FISH-CLSM and viable count of rice roots at 45 DPI of drought-tolerant consortium and after 15 days of water stress imposition in the pot experiment under net house conditions. Treatments; non-inoculated control **(A, E)**, inoculated control **(B, F)**, inoculated stressed **(C, G)**, and non-inoculated stressed **(D, H)**. Determination of plant growth-promoting (PGP) traits of reisolated colonies **(I)**. B, bacteria; RC, root cells; CS, inoculated consortium.

A comparison of the bacterial populations in different treatments using FISH showed the presence of more bacteria on inoculated stressed roots as compared with the non-inoculated stressed roots (**Figures 8A–D**). The survival of inoculated drought-tolerant bacteria in the rhizosphere of rice genotypes was studied after the induction of water withholding under net house conditions in a pot experiment. To measure the viable bacterial population, the viable count method was also used at 15 DAS. *Bacillus* sp. NM-2, *Brucella haematophila* NM-4, and *Bacillus* sp. NM-6 were identified from reisolated colonies on the basis of light microscopic studies (**Figures 8E–H**) and PGP traits such as solubilized P (310–353 µg ml^-1^), IAA production (30.8–45.4 µg ml^-1^), and drought tolerance at 20% PEG was checked as compared with the pure colonies (**Figure 8I**).

### 3.7 Effect of the drought-tolerant consortium on rice yield attributes

Consortium inoculation significantly improved the yield and yield-related traits of all the tested rice genotypes under well-irrigated control as compared with non-inoculated control (**Table 5**). Water scarcity reduced the plant height (74–82 cm) as compared with the control (87–101 cm). Inoculation significantly improved the plant height (7%) of V2 under stressed conditions as compared with the non-inoculated stressed plants (**Table 5**). Water stress adversely affected the number of tillers per plant and ultimately the yield.

Inoculation significantly increased (12%) the number of tillers of V2 followed by V3 (11%) and under-stressed as compared with respective non-inoculated stressed plants (**Table 5**). An increase of 33%, 44%, and 87% in plant fresh weight of inoculated V3, V2, and V1, respectively, was observed as compared with non-inoculated stressed plants (**Table 5**). Inoculation significantly increased the grain weight (26%) in V2 plants under stress as compared with non-inoculated stressed plants (**Table 5**).

### 3.8 Principal component analysis

**Figure 9:**
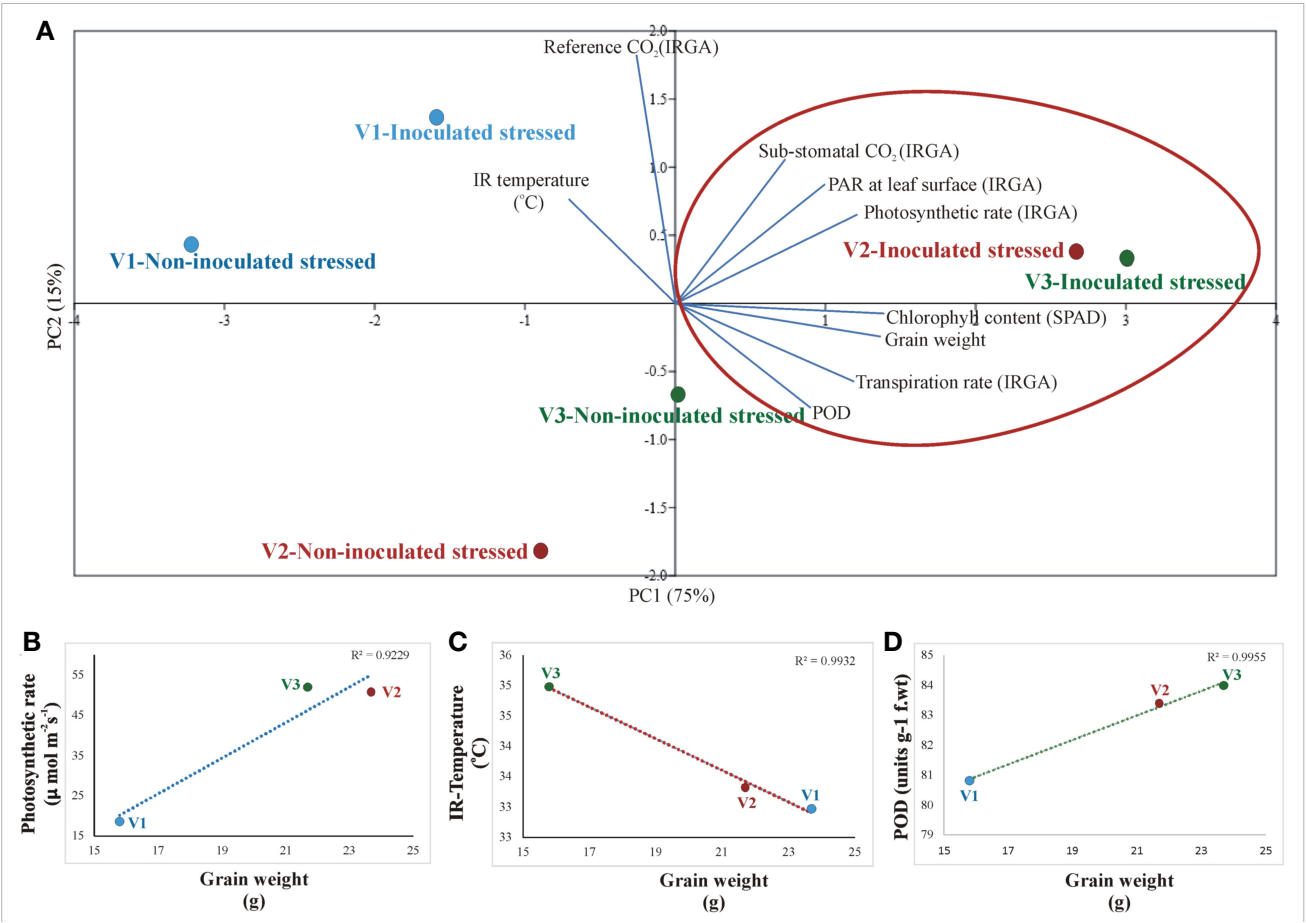
Principal component analysis (PCA) of different instrumental data (IRTI, IRGA, and SPAD) with biochemical parameters across different rice genotypes; V1: Super Basmati, V2: NIBGE-DT02, and V3: IR55419-04 in response to drought-tolerant consortium inoculation and non-inoculation under stress conditions. The points represent mean values of each combination of plant parameters in correlation to different treatments.

A principal component analysis (PCA) was performed to determine the correlation between plant parameters (physio-biochemical, yield, photosynthetic, IRTI, and SPAD values) of inoculated stressed and non-inoculated stressed rice genotypes (**Figure 9**). Principal component analysis (PCA) validated the positive correlation between consortium inoculation with grain yield, photosynthetic rate, chlorophyll content, transpiration rate, sub-stomatal conductance, reference CO_2_, and IR temperature across rice genotypes. The two principal components contributed up to 95% toward variance on the x-axis (PC1 = 75%) and y-axis (PC2 = 15%). No parameter was found to have a negative effect on drought-tolerant inoculation (**Figure 9**).

## 4 Discussion

Water scarcity accompanied by decreased precipitation is one of the key obstacles to agricultural throughput globally and is expected to increase further, hence posing a major threat to future food security (Uzma et al., 2022). Pakistan is the sixth most drought-prone country in the world (UNCCD, 2022). In Pakistan, about 15 million hectares of cultivated area are affected by the aforementioned conditions resulting in less crop productivity and the situation is expected to worsen by the year 2025 (WHO, 2019). According to the Pakistan Rice Sector Report 2020, rice crop area decreased by 3.1% and crop production reduced to 3.3% due to a decrease in cultivated area, water shortage, and dry weather (Anser et al., 2020).

To cope with the adverse effects of global climate change, increasing population, and food insecurity, there is a dire need to develop and screen rice genotypes tolerant to water scarcity with high yield for sustainable rice production (Villalobos-López et al., 2022). Plant growth-promoting bacteria (PGPB) can mitigate the adverse effects of drought on crops (Barquero et al., 2022). Integration of sustainable approaches like plant-associated microbes and drought-tolerant varieties can enhance rice crop production under these alarming conditions of climate change (Kumar and Verma, 2019; Zhang et al., 2020; Shaffique et al., 2022).

Therefore, in the present study rhizospheric soil samples were collected from major rice-growing fields for the isolation of bacteria. The isolated bacterial population was screened for drought tolerance using 20% PEG-8000 and PGP traits (IAA production, P solubilization, organic acid production, EPS, siderophore, and ACC production) (**Table 1**). Recent studies reported that IAA-, EPS-, and ACC-producing bacteria significantly improve the growth and yield of mung bean (Uzma et al., 2022), rice (Zhang et al., 2020), and tomato (Ghosh et al., 2019) under drought stress conditions. Bacteria with a combination of drought stress tolerance and multiple PGP determinants were reported as wheat growth-promoting under well-irrigated and stressed conditions (Zia et al., 2021). Danish and Zafar-ul-Hye (2019) also reported that maximum drought-tolerant strains show ACC deaminase production.

Santoyo et al. (2016) reported that PGPB can promote the activity of ACC deaminase, which directly or indirectly improves plant growth. PGPB can reduce the concentration of ethylene by consuming the ACC before its oxidation through ACC oxidases produced by the plant (Din et al., 2019). Drought-tolerant bacteria, i.e., *E. aerogenes* and *B. thuringiensis*, have been reported as higher phosphate-solubilizing bacteria with ACC deaminase activity (Timmusk et al., 2014; Zhang et al., 2020). Results of the present study showed maximum P solubilization (SI: 5.6, 356 µg ml^-1^) by *Brucella haematophila* NM-4 (**Table 1**). According to Uzma et al., 2022, *Pseudomonas aeruginosa* (MK513748) showed to have maximum P solubilizing capability (95 µg ml^-1^) and solubilization index (SI: 3.3). The potential drought-tolerant rhizobacteria from the present study belonged to the genera *Ochrobactrum* (strains: NM-1), *Brucella* (strains: NM-3, NM-4), and *Bacillus* (strains: NM-2, NM-5, NM-6, NM-7, and NM-8). Ahmad et al., 2022a reported that *Bacillus thuringiensis*, *Bacillus amyloliquefaciens*, and *Ochrobactrum pseudogrignonense* can enhance plant growth of wheat, maize, and rice under water stress.

In the present study, selected drought-tolerant bacteria produced acetic acid, citric acid, gluconic acid, and succinic acid during P solubilization in NBRIP medium as detected by HPLC (**Table 1**). The production of organic acids is one of the key mechanisms involved in P solubilization (Kapadia et al., 2022). Three most efficient drought-tolerant bacteria, i.e., *Bacillus subtilis* NM2, *Brucella haematophila* NM4, and *Bacillus cereus* NM-6, produced higher acetic acid, succinic acid, and gluconic acid (**Table 1**). *Bacillus* sp. and *Enterobacter* sp. solubilized inorganic P and produced different organic acids such as gluconic acid, citric acid, lactic acid, propionic acid, and succinic acid (Sarkar et al., 2018; Mendes et al., 2020).

Multiple strains of the genus *Bacillus* showed a significant role in the alleviation of abiotic stresses in different crops due to their ability to produce IAA, EPS, and siderophore production (Chandra et al., 2018; Din et al., 2019). Concomitant to these findings, the present study showed maximum production of IAA (46.2 µg ml^-1^) by *Brucella haematophila* NM-4 followed by *Bacillus cereus* NM-6 (37.5 µg ml^-1^) and *Bacillus subtilis* NM-2 (31.5 µg ml^-1^) (**Table 1**). Abdelaal et al. (2021) reported that the growth hormone, i.e., IAA, produced by plant growth-promoting bacteria can enhance plant growth, especially the root architecture under stressed conditions. *Bacillus subtilis* NM-2 also showed the production of polysaccharide (fructose), detected by TLC. EPS-producing PGPB enhances soil aggregation and protects plants under water scarcity by retaining increased water potential around the root area (Ojuederie et al., 2019). These isolated bacteria also showed the production of iron-chelating compounds (i.e., siderophores) (**Figure 1**, **Table 1**).

The improved vigor index (1009.2-1100) was observed in response to consortium inoculation under PEG-8000-induced osmotic stress (**Table S2**). *Pseudomonas balearica* RF-2 inoculation showed increased wheat seed germination and seedling vigor under 20% PEG-induced osmotic stress tolerance (Razzaq et al., 2017; Zia et al., 2021). Arun K et al. (2020) also reported that *Bacillus megaterium* PB50 with multifarious PGP determinants showed drought stress alleviating potency in rice plants. Inoculation of *Pseudomonas fluorescence* strains DR7 and DR11 stimulated the seed germination capability of foxtail millet from 13 to 141% over the non-treated seeds under the high level of osmotic stress (-0.5 MPa) (Niu et al., 2018).

Rhizoscanning of seedlings was also carried out to evaluate the effect of drought-tolerant PGPB on root architecture. Likewise, improved root rhizoscanning parameters like root surface area (3.46 cm^2^) and length per volume (36.5 cm m^-3^) were found in consortium-inoculated osmotic stressed seedlings (**Figure 2**; **Table 2**). Previous studies reported that *Bacillus* sp. 12D6 and *Bacillus megaterium* inoculation improved root architecture (root length and diameter), wheat and peanut adaptability, and nutrient uptake under water-stressed conditions (Jochum et al., 2019; Grover et al., 2021).

*In planta* evaluation of the drought-tolerant consortium (*Brucella* sp. NM-4, *Bacillus subtilis* NM-2, and *Bacillus cereus* NM-6) was further carried out in well-irrigated and water-stressed conditions in a pot experiment under net house conditions. The beneficial PGPR associated with the plant roots and tissues reduces abiotic stress on morphological, physiological, and biochemical mechanisms (Shahzad et al., 2013; Rolli et al., 2015). Dubey et al. (2019) reported that plant growth parameters, i.e., root/shoot length, plant fresh and dry weights, were reduced as the water stress increased. These findings supported our results as plant fresh and dry weights of inoculated plants improved as compared with the non-inoculated stressed plants (**Figure 7C**), whereas significant improvement in seedling growth parameters was observed in inoculated stressed plants as compared with the non-inoculated stressed plants (Ali et al., 2022).

Plant growth and productivity are the results of well-regulated photosynthesis, functioning in the leaf and important interlinked physiological responses (Lefe et al., 2017). In the present study, growth inhibition in water-deficit plants might be linked with the rate of photosynthesis, which resulted in stunted plant growth and development. However, consortium inoculation showed an improved plant growth and capability to maintain a higher rate of photosynthesis, the concentration of intracellular CO_2_, transpiration rate, and stomatal conductance as compared with the non-inoculated stressed plants (**Table 3**). Batool et al. (2020) stated that stomatal conductance, rate of transpiration, photosynthesis, and intracellular CO_2_ are interconnected such that under drought stress downregulation in the efficiency of one decreased that of another. Zhang et al., 2022 reported that *Bacillus pumilus* inoculation improved the photosynthetic characteristics (stomatal conductance, photosynthetic and transpiration rates) of *G. uralensis* plants grown under drought stress.

The present study showed stimulation (50%) of relative water content (RWC) in V2-inoculated plants as compared with non-inoculated stressed plants (**Table 5**). Abbasi et al. (2020) reported that *Streptomyces* strains IT25 and C-2012 not only suppressed the signals of stress in tomato plants but also improved plant water status in the form of RWC. Nemeskeri et al. (2022) stated that inoculation of *Pseudomonas* sp. MUS04 in tomato plants improved the water uptake and also decreased the plant temperature as compared with non-inoculated stressed plants. These findings are congruent to the present study, as a percent decrease at 15 DAS was observed in inoculated rice genotype V2, i.e., 6% as compared with non-inoculated stressed plants (**Table 4**).

**Table 5:** Effect of inoculation on yield attributes under water stress in a pot experiment under net house conditions.

Treatments	Genotypes	Plant height^a^ (cm)	No. of tillers per plant^b^	Plant fresh weight^c^	Grain weight^d^ (g)	100 grain weight^e^ (g)
**Non-inoculated control**	V1	100.50 ± 4.8b	10.25 ± 0.5de	58.58 ± 2.8b	28.15 ± 1.8e	1.95 ± 0.1f
	V2	91.75 ± 1.7de	11.75 ± 0.5c	42.90 ± 2.03cd	31.55 ± 0.8cd	2.70 ± 0.29e
	V3	87.25 ± 3.2f	10.70 ± 0.5d	43.68 ± 0.6c	32.45 ± 0.9bc	2.90 ± 0.14e
**Inoculated control**	V1	111.50 ± 3.7a	12.00 ± 0.8c	70.1 ± 2.5a	30.20 ± 1.3d	3.50 ± 0.58d
	V2	97.50 ± 4.2bc	14.00 ± 0.8a	61.13 ± 2.6b	34.45 ± 1.3ab	5.30 ± 0.27b
	V3	93.25 ± 2.9cd	12.73 ± 0.5b	58.65 ± 2.9b	36.30 ± 1a	5.95 ± 0.1a
**Inoculated stressed**	V1	87.75 ± 1.7ef	6.50 ± 0.6g	35.23 ± 1.5e	15.80 ± 0.3h	2.25 ± 0.21f
	V2	88.00 ± 2.16ef	10.20 ± 0.5de	33.00 ± 1.8ef	21.7 ± 0.7fg	3.70 ± 0.36cd
	V3	84.25 ± 1.7fg	9.95 ± 0.2e	40.18 ± 2.1d	23.70 ± 0.9f	4.00 ± 0.2c
**Non-inoculated stressed**	V1	73.75 ± 3.3h	3.98 ± 0.05h	18.85 ± 0.9h	11.45 ± 0.8i	1.37 ± 0.04g
	V2	81.50 ± 1.3g	8.75 ± 0.5f	23.00 ± 0.8g	16.25 ± 0.6h	2.03 ± 0.1f
	V3	80.00 ± 3.7g	8.95 ± 0.05f	30.13 ± 2.01f	20.30 ± 0.7g	2.00 ± 0.1f

Evaluation of inoculation under water stress on yield and yield related attributes:

^a^Plant height (cm),

^b^no. of tillers per plant,

^c^plant fresh weight (g),

^d^grain weight (g), and

^e^100 grain weight (g) of rice genotypes; V1: Super Basmati, V2: NIBGE-DT02, and V3: IR55419-04 under net house conditions. Treatments: non-inoculated control, inoculated control, inoculated stressed, and non-inoculated stressed. Mean data were represented and means are an average of six biological replicates, and each replicate has three plants (10 leaves per plant) in the homogenized sample. Means with the same letter showed non-significant data at p = 0.05, and those with different letters differ significantly according to LSD.

Ali et al. (2022) reported that inoculation of *Bacillus* strains improved the chlorophyll content in drought and salt-stressed rice plants as compared with the non-inoculated stressed plants. However, in the present study in plants inoculated with the drought-tolerant consortium, an increased amount of chlorophyll content was observed (**Table S4, S6**). This increase in chlorophyll content might be the source of improved plant growth under water-deficit conditions (Nadeem et al., 2010; Ahmad et al., 2013; Kang et al., 2014). The present study showed that inoculation with the drought-tolerant plant growth-promoting consortium stimulated stressed plants to accumulate proline content with 122%, 114%, and 85.8% increased rates in three rice genotypes, i.e., V2, V1, and V3, respectively, as compared with non-inoculated stressed plants (**Table 4**). Wu et al. (2014) stated that inoculated plants synthesized and accumulated a significant amount of proline, a compatible solute, to develop osmotic adjustment under water stress. Accumulation of osmoprotectant (proline) is one of the key cellular adaptations that plants undergo for osmotic adjustment during water scarcity (Zouari et al., 2019). Shaffique et al. (2022) reported that PGPB increases the proline content in plants, which minimizes dehydration loss, protects membrane proteins, and maintains cellular turgidity caused by water stress. Dastogeer et al. (2020) reported that the proline content and photosynthetic rate of plants are closely linked, which is consistent with our results, whereas consortium inoculation increased proline content in all tested genotypes under water scarcity, confirming the beneficial role of proline in maintaining photosynthetic activity (Ahmad et al., 2022a).

Increased electrolyte leakage (EL) damages the plant cell membrane under abiotic stress (Gangadhar et al., 2016). The present study showed that the PGPB consortium improved the membrane stability index by 62% of V2 under water stress as compared with the non-inoculated stress plants (**Table 4**). Uzma et al. (2022) reported that plant growth-promoting bacterial inoculation increased the membrane stability of plants under water-stressed conditions as compared with the non-inoculated stressed plants.

In the current study, we found that the consortium-inoculated three rice genotypes (V2, V3, and V1) showed increased accumulation of antioxidants (SOD, CAT, PAL, PPO, and POD) variably under water stress as compared with non-inoculated stressed plants (**Table 4**). Al-Zahrani et al. (2022) reported that plant inoculation with PGPB often reduces the overproduction of ROS by producing more ROS-scavenging enzymes. Antioxidant enzyme production is an effective mechanism, i.e., microbe-induced systemic tolerance (MIST), which confers tolerance to drought (Fadiji et al., 2022). Singh et al. (2020) reported that microbial inoculation activated the antioxidant enzymes PAL and PPO in plants to alleviate oxidative stress and crop yield as compared with the non-inoculated plants.

Consortium inoculation significantly improved the yield and yield-related attributes of all the tested genotypes under well-irrigated treatment (IC) as compared with their well-irrigated controls (**Table 5**). Ilyas et al. (2020) reported that PGPB improves root architecture that helps to enhance plants’ water and nutrient uptake efficiency, resulting in low plant temperature and better growth and yield under water scarcity. Inoculation significantly increased the grain weight (26%) of V2 under water scarcity as compared with non-inoculated water-stressed plants (**Table 5**). Raheem et al. (2018) reported that plant inoculation with drought-tolerant plant growth-promoting bacteria improved the shoot length, the number of grains, and the total yield as compared with their respective control (non-inoculated) stressed plants.

Principal component analysis (PCA) confirmed the positive correlation between drought-tolerant bacterial inoculation with grain yield, photosynthetic rate, chlorophyll content, transpiration rate, sub-stomatal conductance, reference CO_2_, and IR temperature across inoculated varieties (**Figure 9**). No parameter was found to have a negative effect on drought-tolerant bacterial inoculation. Regression analysis further validated the positive correlation between yield, photosynthetic rate, IR temperature, and POD in inoculated rice plants (**Figure 9**).

PGPB used multiple direct and indirect mechanisms, i.e., IAA production, phosphate solubilization, EPS production, ACC deaminase activity, and siderophore production for the alleviation of drought stress and plant growth promotion. Through these mechanisms, rhizobacteria enhance the water-retaining capability of the soil and improve photosynthetic characteristics, water uptake, and nutrient uptake by plants through alteration in root architecture, which in return reduces the plants’ temperature, alleviates ethylene stress in plants, and enhances cellular turgor and osmotic adjustment through the accumulation of osmoprotectant (proline) under water-deficit conditions. Hence, as a result of this, the beneficial interaction between drought-tolerant PGPB and plants improved the crop yield under water scarcity.

In Pakistan, Basmati rice with aromatic long grain has significant importance among rice traders, millers, and consumers. It covers more than 80% of the total Basmati area in the major rice-growing province Punjab, but it is susceptible to water stress (Sabar et al., 2019). The current scenario of the climatic shift resulted in uncertainty for rice growers due to water shortage. In the present study, consortium inoculation showed its supporting role to sustain the yield of rice genotypes NIBGE-DT02 and Super Basmati under water scarcity. The integration of drought-tolerant bioinoculant and genotype (NIBGE-DT02) will lead to improved water stress tolerance concomitant with the increased yield under alarming conditions of climate change. Based on the promising results of the designed consortium, whole-genome sequencing of the promising drought-tolerant plant growth-promoting bacteria (*Bacillus subtilis* NM-2, *Brucella haematophilum* NM-4, and *Bacillus cereus* NM-6) was performed (unpublished data) and is currently being annotated and studied for genes and multiple pathways involved in drought tolerance. This will allow more insight into the genetics behind the synergism between the drought-tolerant bacteria and pants to alleviate and survive under severe water stress.

## 5 Conclusion

Exploiting the beneficial consortium as bioinoculant and drought-tolerant rice genotypes can efficaciously enhance crop productivity under water scarcity. Novel strains *Bacillus subtilis* NM-2, *Brucella haematophilum* NM-4, and *Bacillus cereus* NM-6 that can produce EPS, IAA, siderophore, ACC deaminase, and organic acids and solubilize P were drought-tolerated up to -0.5 MPa (20% PEG-8000). The developed consortium showed the efficient ability to alleviate drought stress in the rice genotype with significantly improved grain yield. Furthermore, bacterial inoculation will be investigated to explore the real-time expression of drought-responsive genes in rice plants under osmotic stress. Hence, in the future, integrated application of these potential drought-tolerant bacteria along with drought-tolerant rice genotypes and advanced non-destructive, handheld robust instruments, i.e., infrared thermal imaging (IRTI) and infrared gas analyzer (IRGA), with conventional methods will be used for the efficient selection and timely prevention of yield loss from water scarcity.

## Data availability statement

The datasets presented in this study can be found in online repositories. The names of the repository/repositories and accession number(s) can be found in the article/**Supplementary Material**.

## Author contributions

NM did the overall execution of the experiment, analytical work, collection of IRGA data, infrared thermal images and software analysis, collection of data after morpho-physiological and biochemical analysis of leaves, organization of resulting data, and writing up and revision of manuscript. MAr and SYa contributed to the planning, designing and finalization of basic idea of experiments and overall supervision during analytical work, revised and finalized the manuscript. MAs did the arrangement and provision of rice seeds, contributed in study basic idea and planning of net house experiment and help in data collection. MY and KE performed data analysis and reviewed the manuscript. SYo helped in plant antioxidant analysis and data execution. M-u-R and SZ provide the Infrared Gas Analyzer and helped in the data collection and analysis. IA helped in the sequencing of 16S rRNA gene amplification. AI helped in FISH analysis and SK helped exopolysaccharide detection. All authors contributed to the article and approved the submitted version.

## Supplementary material

Supplementary Figure 1 Total colony forming units (CFU) obtained at different concentrations of Polyethylene Glycol (PEG)-induced stress in LB medium for isolation of drought tolerant bacteria. FB: Faisalabad, SK: Sialkot, SP: Sheikhupura, HF: Hafizabad, PB: Pindi Bhattian, NN: Nankana Sahib, NM: Narang Mandi, GW: Gujranwala and FA: Farooqabad.

Supplementary Figure 2 Phylogenetic tree based on 16S rRNA sequencing of drought-tolerant bacteria

Supplementary Figure 3 pH change at 7^th^ day of post inoculation (DPI) by rice rhizosphere drought tolerant bacteria in NBRIP′s broth having tricalcium phosphate (TCP) as insoluble phosphate source.

## References

[B1] Aalipour H. Nikbakht A. Etemadi N. Rejali F. Soleimani M. (2020). Biochemical response and interactions between arbuscular mycorrhizal fungi and plant growth promoting rhizobacteria during establishment and stimulating growth of Arizona cypress (*Cupressus arizonica* g.) under drought stress. Sci. Hortic. 261, 108–923. doi: 10.1016/j.scienta.2019.108923

[B2] Abbasi S. Sadeghi A. Safaie N. (2020). *Streptomyces* alleviate drought stress in tomato plants and modulate the expression of transcription factors ERF1 and WRKY70 genes. Sci. Hortic. 265, 109206. doi: 10.1016/j.scienta.2020.109206

[B3] Abdelaal K. AlKahtani M. Attia K. Hafez Y. Kiraly L. Künstler A. (2021). The role of plant growth-promoting bacteria in alleviating the adverse effects of drought on plants. Biology 10, 520. doi: 10.3390/biology10060520 34207963 PMC8230635

[B4] Ahmad H. M. Fiaz S. Hafeez S. Zahra S. Shah A. N. Gul B.. (2022a). Plant growth promoting rhizobacteria eliminate the effect of drought stress in plants: a review. Front. Plant Sci. 19, 65. doi: 10.3389/fpls.2022.875774 PMC9406510 36035658

[B5] Ahmad H. Zafar S. A. Naeem M. K. Shokat S. Inam S. Naveed S. A.. (2022b). Impact of pre-anthesis drought stress on physiology, yield-related traits, and drought-responsive genes in green super rice. Front. Genet. 256. doi: 10.1101/2021.11.18.469071 PMC8987348 35401708

[B6] Ahmad M. Zahir Z. A. Khalid M. Nazli F. Arshad M. (2013). Efficacy of rhizobium and pseudomonas strains to improve physiology, ionic balance and quality of mung bean under salt-affected conditions on farmer′s fields. Plant Physiol. Biochem. 63, 170–176. doi: 10.1016/j.plaphy.2012.11.024 23262185

[B7] Ali Q. Ayaz M. Mu G. Hussain A. Yuanyuan Q. Yu C.. (2022). Revealing plant growth-promoting mechanisms o*f bacillus* strains in elevating rice growth and its interaction with salt stress. Front. Plant Sci. 13, 994902. doi: 10.3389/fpls.2022.994902 36119605 PMC9479341

[B8] Ali S. Z. Sandhya V. Rao L. V. (2014). Isolation and characterization of drought-tolerant ACC deaminase and exopolysaccharide-producing fluorescent *Pseudomonas* sp. Ann. Microbiol. 64, 493–502. doi: 10.1007/s13213-013-0680-3

[B9] Al-Zahrani H. S. Alharby H. F. Fahad S. (2022). Antioxidative defense system, hormones, and metabolite accumulation in different plant parts of two contrasting rice cultivars as influenced by plant growth regulators under heat stress. Front. Plant Sci. 13. doi: 10.3389/fpls.2022.911846 PMC9196032 35712584

[B10] Amann R. I. Ludwig W. Schleifer K. H. (1995). Phylogenetic identification and *in situ* detection of individual microbial cells without cultivation. Microbiol. Rev. 59, 143–169. doi: 10.1128/mr.59.1.143-169.1995 7535888 PMC239358

[B11] Ames-Gottfred N. P. Christie B. R. Jordan D. C. (1989). Use of the chrome azurol s agar plate technique to differentiate strains and field isolates of *Rhizobium leguminosarum* biovar *trifilii* . Appl. Environ. Microbiol. 55, 707–710. doi: 10.1128/aem.55.3.707-710.1989 16347877 PMC184184

[B12] Anser M. K. Hina T. Hameed S. Nasir M. H. Ahmad I. Naseer M. A. U. R. (2020). Modeling adaptation strategies against climate change impacts in integrated rice-wheat agricultural production system of Pakistan. Int. J. Environ. Res.Public Health 7, 2522. doi: 10.3390/ijerph17072522 PMC7177414 32272663

[B13] Anwar M. A. Kralj S. Pique A. V. Leemhuis H. van der Maarel M. J. Dijkhuizen L. (2010). Inulin and levan synthesis by probiotic lactobacillus gasseri strains: Characterization of three novel fructansucrase enzymes and their fructan products. Microbiology 156, 1264–1274. doi: 10.1099/mic.0.036616-0 20075040

[B14] Arun K D. Sabarinathan K. G. Gomathy M. Kannan R. Balachandar D. (2020). Mitigation of drought stress in rice crop with plant growth-promoting abiotic stress-tolerant rice phyllosphere bacteria. J.Basic Microbiol. 9, 768–786. doi: 10.1002/jobm.202000011 32667057

[B15] Ashraf M. Y. Akhtar K. Hussain F. Iqbal J. (2006). Screening of different accessions of three potential grass species from cholistan desert for salt tolerance. Pak. J. Bot. 38, 1589–1597.

[B16] (2018) World health organization, Pakistan: Drought in sindh and Baluchistan, 469 situation report (Accessed 2019).

[B17] Barquero M. Poveda J. Laureano-Marín A. M. Ortiz-Liébana N. Brañas J. González-Andrés F. (2022). Mechanisms involved in drought stress tolerance triggered by rhizobia strains in wheat. Front. Plant Sci. 13. doi: 10.3389/fpls.2022.1036973 PMC9686006 36438093

[B18] Bates L. S. Waldren R. P. Teare I. D. (1973). Rapid determination of free proline for water-stress studies. Plant Soil 39, 205–207. doi: 10.1007/BF00018060

[B19] Batool T. Ali S. Seleiman M. F. Naveed N. H. Ali A. Ahmed K.. (2020). Plant growth promoting rhizobacteria alleviates drought stress in potato in response to suppressive oxidative stress and antioxidant enzymes activities. Sci. Rep. 10, 1–19. doi: 10.1038/s41598-020-73489-z 33046721 PMC7550571

[B20] Bernal E. Rotondo F. Roman-Reyna V. Klass T. Timilsina S. Minsavage G. V.. (2022). Migration drives the replacement of xanthomonas perforans races in the absence of widely deployed resistance. Front. Microbiol. 13. doi: 10.3389/fmicb.2022.826386 PMC8971904 35369455

[B21] Chance B. Maehly A. (1955). Assay of catalase and peroxidase. Methods Enzymol. 2, 764–817. doi: 10.1016/S0076-6879(55)02300-8

[B22] Chandra D. Srivastava R. Glick B. R. Sharma A. K. (2018). Drought-tolerant pseudomonas spp. improve the growth performance of finger millet (*Eleusine coracana* (L.) under non-stressed and drought stressed conditions. Pedosphere 28, 227–240. doi: 10.1016/S1002-0160(18)60013-X

[B23] Chen D. Wang S. Cao B. Cao D. Leng G. Li H.. (2016). Genotypic variation in growth and physiological response to drought stress and re-watering reveals the critical role of recovery in drought adaptation in maize seedlings. Front. Plant Sci. 6, 12–41. doi: 10.3389/fpls.2015.01241 PMC4709455 26793218

[B24] Danish S. Zafar-ul-Hye M. (2019). Co-Application of ACC-deaminase producing PGPR and timber-waste biochar improves pigments formation, growth and yield of wheat under drought stress. Sci. Rep. 9, 1–13. doi: 10.1038/s41598-018-37186-2 30979925 PMC6461675

[B25] Dastogeer K. M. G. Chakraborty A. Sarker M. S. A. Akter M. A. (2020). Roles of fungal endophytes and viruses in mediating drought stress tolerance in plants. Int. J. Agricult. Biol. 24, 1497–1512. doi: 10.17957/IJAB/15.1588

[B26] Delshadi S. Ebrahimi M. Shirmohammadi E. (2017). Influence of plant-growth-promoting bacteria on germination, growth and nutrients′ uptake of *Onobrychis sativa* l. under drought stress. J. Plant Interact. 12, 200–208. doi: 10.1080/17429145.2017.1316527

[B27] Deng L. Peng C. Kim D. G. Li J. Liu Y. Hai X.. (2021). Drought effects on soil carbon and nitrogen dynamics in global natural ecosystems. Earth Sci. Rev. 214, 103501. doi: 10.1016/j.earscirev.2020.103501

[B28] Din B. U. Sarfraz S. Xia Y. Kamran M. A. Javed M. T. Sultan T.. (2019). Mechanistic elucidation of germination potential and growth of wheat inoculated with exopolysaccharide and ACC-deaminase producing bacillus strains under induced salinity stress. Ecotoxicol. Environ. Saf. 183, 109–466. doi: 10.1016/j.ecoenv.2019.109466 31408821

[B29] Duan B. Li L. Chen G. Su-Zhou C. Li Y. Merkeryan H.. (2021). 1-Aminocyclopropane-1-Carboxylate deaminase-producing plant growth-promoting rhizobacteria improve drought stress tolerance in grapevine (*Vitis vinifera* l.). Front. Plant Sci. 12, 12, 706–990. doi: 10.3389/fpls.2021.706990 PMC10305780 37388278

[B30] Dubey A. Kumar A. Abd-Allah E. F. Hashem A. Khan M. L. (2019). Growing more with less: breeding and developing drought resilient soybean to improve food security. Ecol. Indic. 105, 425–437. doi: 10.1016/j.ecolind.2018.03.003

[B31] El-Mageed A. Taia A. El-Mageed A. Shimaa A. El-Saadony M. T. Abdelaziz S.. (2022). Plant growth-promoting rhizobacteria improve growth, morph-physiological responses, water productivity, and yield of rice plants under full and deficit drip irrigation. Rice 15, 1–15. doi: 10.1186/s12284-022-00564-6 35288814 PMC8921367

[B32] Fadiji A. E. Santoyo G. Yadav A. N. Babalola O. O. (2022). Efforts towards overcoming drought stress in crops: Revisiting the mechanisms employed by plant growth-promoting bacteria. Front. Microbiol. 13. doi: 10.3389/fmicb.2022.962427 PMC9372271 35966701

[B33] Gangadhar B. H. Sajeesh K. Venkatesh J. Gene S. (2016). Enhanced tolerance of transgenic potato plants over-expressing non-specific lipid transfer protein-1 (StnsLTP1) against multiple abiotic stresses isolation and gateway cloning of. Front. Plant Sci. 7, 1–12. doi: 10.3389/fpls.2016.01228 27597854 PMC4993012

[B34] Ghosh D. Gupta A. Mohapatra S. (2019). A comparative analysis of exopolysaccharide and phytohormone secretions by four drought-tolerant rhizobacterial strains and their impact on osmotic-stress mitigation in *Arabidopsis thaliana* . World J. Microbiol. Biotechnol. 35, 90. doi: 10.1007/s11274-019-2659-0 31147784

[B35] Giaanopolitis N. Ries S. (1977). Superoxide dismutase I, occurrence in higher plants. Plant Physio1. 59, 309–314. doi: 10.1104/pp.59.2.309 PMC542387 16659839

[B36] Global times (2022). China To ramp up coal supply for power generation amid prolonged heat waves by global times.

[B37] Gordon S. A. Weber R. P. (1951). Colorimetric estimation of indoleacetic acid. Plant Physiol. 26, 192–195. doi: 10.1104/pp.26.1.192 16654351 PMC437633

[B38] Grover M. Bodhankar S. Sharma A. Sharma P. Singh J. Nain L. (2021). PGPR mediated alterations in root traits: way towards sustainable crop production. Front. Sustain. Food Syst. 4. doi: 10.3389/fsufs.2020.618230

[B39] Ilyas N. Mumtaz K. Akhtar N. Yasmin H. Sayyed R. Z. Khan W.. (2020). Exopolysaccharides producing bacteria for the amelioration of drought stress in wheat. Sustainability 12, 76–88. doi: 10.3390/su12218876

[B40] Imran A. Hafeez F. Y. Fruhling A. Schumann P. Malik K. Stackebrandt E. (2010). *Ochrobactrum ciceri* spp. nov., isolated from nodules of *Cicer arietinum* . Int. J. Syst. Evol. Microbiol. 60, 1548–1553. doi: 10.1099/ijs.0.013987-0 19684324

[B41] Inbaraj M. P. (2021). Plant-microbe interactions in alleviating abiotic stress - a mini review. Front. Agron. 28. doi: 10.3389/fagro.2021.667903

[B42] Islam S. Akanda A. M. Prova A. Islam M. T. Hossain M. M. (2016). Isolation and identification of plant growth promoting rhizobacteria from cucumber rhizosphere and their effect on plant growth promotion and disease suppression. Front. Microbiol. 6. doi: 10.3389/fmicb.2015.00006 PMC4735380 26869996

[B43] Jochum M. D. McWilliams K. L. Borrego E. J. Kolomiets M. V. Niu G. Pierson E. A.. (2019). Bioprospecting plant growth-promoting rhizobacteria that mitigate drought stress in grasses. Front. Microbiol. 10. doi: 10.3389/fmicb.2019.02106 PMC6747002 31552009

[B44] JRC. (2022). Drought in Europe august 2022, publications office of the European union, luxembourg 2022. Report of the joint research centre (JRC) 130493, the European commission′s science and knowledge service. doi: 10.2760/264241

[B45] Kang S. M. Khan A. L. Waqas M. You Y. H. Kim J. H. Kim J. G.. (2014). Plant growth-promoting rhizobacteria reduce adverse effects of salinity and osmotic stress by regulating phytohormones and antioxidants in *Cucumis sativus* . J. Plant Interact. 9, 82–673. doi: 10.1080/17429145.2014.894587

[B46] Kannepalli A. Davranov K. Narimanov A. Enakiev Y. Syed S. Elgorban A. M.. (2020). Co-Inoculation of rhizobacteria promotes growth, yield, and nutrient contents in soybean and improves soil enzymes and nutrients under drought conditions. Sci. Rep. 11, 81–220. doi: 10.1038/s41598-021-01337-9 PMC8586231 34764331

[B47] Kapadia C. Patel N. Rana A. Vaidya H. Alfarraj S. Ansari M. J.. (2022). Evaluation of plant growth-promoting and salinity ameliorating potential of halophilic bacteria isolated from saline soil. Front. Plant Sci. 13. doi: 10.3389/fpls.2022.946217 PMC9335293 35909789

[B48] Khairnar M. Hagir A. Parmar K. Sayyed R. James E. Rahi P. (2022). Phylogenetic diversity and plant growth-promoting activities of rhizobia nodulating fenugreek (*Trigonella foenumgraecum* l.) cultivated in different agroclimatic regions of India. FEMS Microbiol. Ecol. 98, 1–13. doi: 10.1093/femsec/fiac014 35142840

[B49] Kim S. L. Kim N. Lee H. Lee E. Cheon K. S. Kim M.. (2020). High-throughput phenotyping platform for analyzing drought tolerance in rice. Planta 252, 1–18. doi: 10.1007/s00425-020-03436-9 32779032 PMC7417419

[B50] Kumar S. Stecher G. Tamura K. (2016). MEGA7: molecular evolutionary genetics analysis version 7.0 for bigger datasets. Mol. Biol. Evol. 33, 1870–1874. doi: 10.1093/molbev/msw054 27004904 PMC8210823

[B51] Kumar A. Verma J. P. (2019). “The role of microbes to improve crop productivity and soil health,” in Ecological wisdom inspired restoration engineering. Eds. Achal V. Mukherjee A. (Berlin: Springer), 249–265.

[B52] Lefe I. Legay S. Lamoureux D. (2017). Identification of drought-responsive compounds in potato through a combined transcriptomic and targeted metabolite approach. J. Exp. Bot. 61, 2327–2343. doi: 10.1093/jxb/erq060 20406784

[B53] Li X. Sun P. Zhang Y. Jin C. Guan C. (2020). A novel PGPR strain kocuria rhizophila Y1 enhances salt stress tolerance in maize by regulating phytohormone levels, nutrient acquisition, redox potential, ion homeostasis, photosynthetic capacity and stress-responsive genes expression. Environ. Exp. Bot. 174, 104–123. doi: 10.1016/j.envexpbot.2020.104023

[B54] Macias-Benitez S. Garcia-Martinez A. M. Caballero Jimenez P. Gonzalez J. M. Tejada Moral M. Parrado Rubio J. (2020). Rhizospheric organic acids as biostimulants: monitoring feedbacks on soil microorganisms and biochemical properties. Front. Plant Sci. 11, 633. doi: 10.3389/fpls.2020.00633 32547578 PMC7270406

[B55] Mahreen N. Yasmin S. Asif M. Yousaf S. Yahya M. Ejaz K.. (2022). Integrated analysis of osmotic stress and infrared thermal imaging for the selection of resilient rice under water scarcity. Front. Plant Sci. 13. doi: 10.3389/fpls.2022.834520 PMC8882677 35237292

[B56] Manz W. Amann R. Ludwig W. Vancanneyt M. Schleifer K. H. (1996). Application of a suite of 16S rRNA-specific oligonucleotide probes designed to investigate bacteria of the phylum cytophaga-flavobacter-bacteroides in the natural environment. Microbiol. 142, 1097–1106. doi: 10.1099/13500872-142-5-1097 8704951

[B57] Mathur S. Tomar R. S. Jajoo A. (2018). Arbuscular mycorrhizal fungi (AMF) protects photosynthetic apparatus of wheat under drought stress. Photosyn. Res. 39, 227–238. doi: 10.1007/s11120-018-0538- 29982909

[B58] Mendes G. de O. Murta H. M. Valadares R. V. Silveira W. B. da Silva I. R.. (2020). Oxalic acid is more efficient than sulfuric acid for rock phosphate solubilization. Miner. Eng. 155, 106–458. doi: 10.1016/j.mineng.2020.106458

[B59] Michel B. E. Kaufmann M. R. (1973). The osmotic potential of polyethylene glycol 6000. Plant Physiol. 51, 914–916. doi: 10.1104/pp.51.5.914 16658439 PMC366375

[B60] Mogrovejo D. C. Perini L. Gostincar C. Sepcic K. Turk M. Ambrozic-Avgustin J.. (2020). Prevalence of antimicrobial resistance and hemolytic phenotypes in culturable arctic bacteria. Front. Microbiol. 11, 570. doi: 10.3389/fmicb.2020.00570 32318045 PMC7147505

[B61] Motaleb K. Z. M. Sharif-ul-Islam M. Hoque M. B. (2018). Improvement of physio-mechanical properties of pineapple leaf fiber reinforced composite. Int. J. Biomater. 73, 84–360. doi: 10.1155/2018/7384360 PMC6020629 30008747

[B62] Mumtaz M. Z. Saqib M. Abbas G. Akhtar J. Ul-Qamar Z. (2019). Drought stress impairs grain yield and quality of rice genotypes by impaired photosynthetic attributes and K nutrition. Rice Sci. 27, 5–9. doi: 10.1016/j.rsci.2019.12.001

[B63] Munawar W. Hameed A. Khan M. K. R. (2021). Differential morphophysiological and biochemical responses of cotton genotypes under various salinity stress levels during early growth stage. Front. Plant Sci. 12, 309–622. doi: 10.3389/fpls.2021.622309 PMC7990906 33777064

[B64] Murphy J. Riley J. (1962). Colorimetric method for determination of p in soil solution. Anal. Chim. Acta 27, 31–36. doi: 10.1016/S0003-2670(00)88444-5

[B65] Murtaza H. Asghari B. Hassan S. G. Javed I. Umer A. Khan K. A. (2014). Enhancement of rice growth and production of growth-promoting phytohormones by inoculation with rhizobium and other rhizobacteria. World Appl. Sci. J. 31, 1734–1743. doi: 10.5829/idosi.wasj.2014

[B66] Nadeem S. M. Zahir Z. A. Naveed M. Asghar H. N. Arshad M. (2010). Rhizobacteria capable of producing ACC-deaminase may mitigate salt stress in wheat. Soil Sci. Soc Am. J. 74, 533–542. doi: 10.2136/sssaj2008.0240

[B67] Nasir A. Sattar F. Ashfaq I. Lindemann S. R. Chen M. H. Van den Ende W.. (2020). Production and characterization of a high molecular weight levan and fructooligosaccharides from a rhizospheric isolate of *Bacillus aryabhattai* . LWT 123, 109093. doi: 10.1016/j.lwt.2020.109093

[B68] Nautiyal C. S. (1999). An efficient microbiological growth medium for screening phosphate solubilizing microorganisms. FEMS Microbiol. Lett. 170, 265–270. doi: 10.1111/j.1574-6968.1999.tb13383.x 9919677

[B69] Nawaz A. Shahbaz M. Imran A. Marghoob M. U. Imtiaz M. Mubeen F. (2020). Potential of salt tolerant PGPR in growth and yield augmentation of wheat (*Triticum aestivum* l.) under saline conditions. Front. Microbiol. 11. doi: 10.3389/fmicb.2020.02019 PMC7562815 33117299

[B70] Nemeskeri E. Horvath K. Z. Andryei B. Ilahy R. Takacs S. Nemenyi A.. (2022). Impact of plant growth-promoting rhizobacteria inoculation on the physiological response and productivity traits of field-grown tomatoes in Hungary. Horticulturae 8, 641. doi: 10.3390/horticulturae8070641

[B71] Nemeskeri E. Nemenyi A. Bocs A. Pek Z. Helyes L. (2019). Physiological factors and their relationship with the productivity of processing tomato under different water supplies. Water 11, 586. doi: 10.3390/w11030586

[B72] Ngumbi E. Kloepper J. (2016). Bacterial-mediated drought tolerance: current and future prospects. Appl. Soil Ecol. 105, 109–125. doi: 10.1016/j.apsoil.2016.04.009

[B73] Niu X. Song L. Xiao Y. Ge W. (2018). Drought-tolerant plant growth-promoting rhizobacteria associated with foxtail millet in a semi-arid agroecosystem and their potential in alleviating drought stress. Front. Microbiol. 8, 25–80. doi: 10.3389/fmicb.2017.02580 PMC5771373 29379471

[B74] Ojuederie O. B. Olanrewaju O. S. Babalola O. O. (2019). Plant growth promoting rhizobacterial mitigation of drought stress in crop plants: implications for sustainable agriculture. Agronomy 9, 712. doi: 10.3390/agronomy9110712

[B75] Passari A. K. Mishra V. K. Singh G. Singh P. Kumar B. Gupta V. K.. (2017). Insights into the functionality of endophytic actinobacteria with a focus on their biosynthetic potential and secondary metabolites production. Sci. Rep. 7, 1–17. doi: 10.1038/s41598-017-12235-4 28924162 PMC5603540

[B76] Penrose D. M. Glick B. R. (2003). Methods for isolating and characterizing ACC deaminase-containing plant growth-promoting rhizobacteria. Physiol. Plant 118, 10–15. doi: 10.1034/j.1399-3054.2003.00086.x 12702008

[B77] Raheem A. Shaposhnikov A. Belimov A. A. Dodd I. C. Ali B. (2018). Auxin production by rhizobacteria was associated with improved yield of wheat (*Triticum aestivum* l.) under drought stress. Arch. Agron. Soil Sci. 64, 574–587. doi: 10.1080/03650340.2017.1362105

[B78] Razzaq H. Tahir N. Hammad M. Sadaqat A. H. Sadia B. (2017). Screening of sunflower (*Helianthus annus* l.) accessions under drought stress conditions, an experimental assay. J. Soil Sci. Plant Nutr. 17, 662–671. doi: 10.4067/S0718-95162017000300009

[B79] Rehman S. U. Bilal M. Rana R. M. Tahir M. N. Shah M. K. N. Ayalew H.. (2016). Cell membrane stability and chlorophyll content variation in wheat (*Triticum aestivum*) genotypes under conditions of heat and drought. Crop Pasture Sci. 67, 712–718. doi: 10.1071/CP15385

[B80] Rolli E. Marasco R. Vigani G. Ettoumi B. Mapelli F. Deangelis M. L.. (2015). Improved plant resistance to drought is promoted by the root-associated microbiome as a water stress-dependent trait. Environ. Microbiol. 17, 316–331. doi: 10.1111/1462-2920.12439 24571749

[B81] Sabar M. Arif M. (2014). Phenotypic response of rice (Oryza sativa) genotypes to variable moisture stress regimes. Int. J. Agric. Biol. 16, 32–40.

[B82] Sabar M. Shabir G. Shah S. M. Aslam K. Naveed S. A. Arif M. (2019). Identification and mapping of QTLs associated with drought tolerance traits in rice by a cross between super basmati and IR55419-04. Breed. Sci. 69, 169–178. doi: 10.1270/jsbbs.18068 31086495 PMC6507710

[B83] Sagar A. Sayyed R. Z. Ramteke P. W. Sharma S. Marraiki N. Elgorban A. M.. (2020). ACC deaminase and antioxidant enzymes producing halophilic enterobacter sp. PR14 promotes the growth of rice and millets under salinity stress. Physiol. Mol. Biol. Plants 26, 1847–1854. doi: 10.1007/s12298-020-00852-9 32943820 PMC7468042

[B84] Santoyo G. Moreno-Hagelsieb G. Del Carmen Orozco-Mosqueda M. Glick B. R. (2016). Plant growth-promoting bacterial endophytes. Microbiol. Res. 183, 92–99. doi: 10.1016/j.micres.2015.11.008 26805622

[B85] Sarkar A. Ghosh P. K. Pramanik K. Mitra S. Soren T. Pandey S.. (2018). A halotolerant enterobacter sp. displaying ACC deaminase activity promotes rice seedling growth under salt stress. Res. Microbiol. 169, 20–32. doi: 10.1016/j.resmic.2017.08.005 28893659

[B86] Shaffique S. Khan M. A. Imran M. Kang S. M. Park Y. S. Wani S. H.. (2022). Research progress in the field of microbial mitigation of drought stress in plants. Front. Plant Sci. 13. doi: 10.3389/fpls.2022.870626 PMC9161204 35665140

[B87] Shahzad S. M. Arif M. S. Riaz M. Iqbal Z. Ashraf M. (2013). PGPR with varied ACC- deaminase activity induced different growth and yield response in maize (*Zea mays* l.) under fertilized conditions. Eur. J. Soil Biol. 57, 27–34. doi: 10.1016/j.ejsobi.2013.04.002

[B88] Shanware A. S. Kalkar S. A. Trivedi M. M. (2014). Potassium solublisers: occurrence, mechanism and their role as competent biofertilizers. Int. J. Curr. Microbiol. Appl. Sci. 3, 622–629.

[B89] Singh D. P. Singh V. Gupta V. K. Shukla R. Prabha R. Sarma B. K.. (2020). Microbial inoculation in rice regulates antioxidative reactions and defense related genes to mitigate drought stress. Sci. Rep. 10, 4818. doi: 10.1038/s41598-020-61140-w 32179779 PMC7076003

[B90] Soltanpour P. Workman S. (1979). Modification of the NH4HCO3-DTPA soil test to omit carbon black. Commun. Soil Sci. Plant Anal. 10, 1411–1420. doi: 10.1080/00103627909366996

[B91] Somasegaran P. Hoben H. J. (1994). Handbook for rhizobia (New York, NY: Springer), 58–64.

[B92] Tien T. M. Gaskins M. H. Hubbell D. H. (1979). Plant growth substances produced by azospirillum brasilense and their effect on the growth of pearl millet (*Pennisetum americanum* l.). Appl. Environ. Microbiol. 6, 1016–1024. doi: 10.1128/aem.37.5.1016-1024.1979 PMC243341 16345372

[B93] Timmusk S. Abd El-Daim I. A. Copolovici L. Tanilas T. Kännaste A. Behers L.. (2014). Drought-tolerance of wheat improved by rhizosphere bacteria from harsh environments: enhanced biomass production and reduced emissions of stress volatiles. PloS One 9, 1–13. doi: 10.1371/journal.pone.0096086 PMC4014485 24811199

[B94] Tovar M. J. Romero M. P. Girona J. Motilva M. J. (2002). L-phenylalanine ammonia-lyase and concentration of phenolics in developing olive (*Olea europaea* l. cv. arbequina) fruit grown under different irrigation regimes. J. Sci. Food. Agric. 82, 892–898. doi: 10.1002/jsfa.1122

[B95] UNCCD (2022). “United nations convention to combat desertification programme 2022,” in The united nations global land outlook report 2022: Dawn, (Abidjan: Dawn news).

[B96] Uzma M. Iqbal A. Hasnain S. (2022). Drought tolerance induction and growth promotion by indole acetic acid producing *Pseudomonas aeruginosa* in *Vigna radiata* . PloS One 17, 262–932. doi: 10.1371/journal.pone.0262932 PMC8815908 35120147

[B97] Villalobos-López M. A. Arroyo-Becerra A. Quintero-Jiménez A. Iturriaga G. (2022). Biotechnological advances to improve abiotic stress tolerance in crops. Int. J. Mol. Sci. 23, 34–39. 10.3390/ijms231912053 PMC9570234 36233352

[B98] Vos P. Garrity G. Jones D. Krieg N. R. Ludwig W. Rainey F. A.. (2011). “Bergey′s manual of systematic bacteriology: Volume 3: The firmicutes (Vol. 3),” in Science and business media (Berlin: Springer).

[B99] Vyas P. Gulati A. (2009). Organic acid production *in vitro* and plant growth promotion in maize under controlled environment by phosphate-solubilizing fluorescent pseudomonas. BMC Microbiol. 9, 174. doi: 10.1186/1471-2180-9-174 19698133 PMC2738680

[B100] Wang H. Tang X. Wang H. Shao H. B. (2015). Proline accumulation and metabolism-related genes expression profiles in *Kosteletzkya virginica* seedlings under salt stress. Front. Plant Sci. 6, 792. doi: 10.3389/fpls.2015.00792 26483809 PMC4586422

[B101] WHO (2019) World health organization Pakistan: Drought in Sindh and Baluchistan, 469 Situation report. (Accessed 2019).

[B102] World facts (2018) The world factbook CIA world factbook (Library of Congress Country Studies, US Department of State) (Accessed 2019).

[B103] Wu S. Hu C. Tan Q. Nie Z. Sun X. (2014). Effects of molybdenum on water utilization, antioxidative defense system and osmotic-adjustment ability in winter wheat (*Triticum aestivum*) under drought stress. Plant Physiol. Biochem. 83, 365–374. doi: 10.1016/j.plaphy.2014.08.022 25221925

[B104] Yahya M. Rasul M. Farooq I. Mahreen N. Tawab A. Irfan M.. (2021). Differential root exudation and architecture for improved growth of wheat mediated by phosphate solubilizing bacteria. Front. Microbiol. 2713. doi: 10.3389/fmicb.2021.744094 PMC8554232 34721342

[B105] Yasmin S. Zaka A. Imran A. Zahid M. A. Yousaf S. Rasul G.. (2016). Plant growth promotion and suppression of bacterial leaf blight in rice by inoculated bacteria. PloS One 11, 13–71. doi: 10.1371/journal.pone.0160688 PMC4988697 27532545

[B106] Yuan Z. Cao Q. Zhang K. Ata-ul-Karim S. T. Tian Y. Zhu Y.. (2016). Optimal leaf positions for SPAD meter measurement in rice. Front. Plant Sci. 7, 719. doi: 10.3389/fpls.2016.00719 27303416 PMC4880590

[B107] Zahid A. Ali S. Ahmed M. Iqbal N. (2020). Improvement of soil health through residue management and conservation tillage in rice-wheat cropping system of punjab, Pakistan. Agronomy 10, 18–44. doi: 10.3390/agronomy10121844

[B108] Zahra S. Hussain M. Zulfiqar S. Ishfaq S. Shaheen T. Akhtar M. (2021). EMS-based mutants are useful for enhancing drought tolerance in spring wheat. Cereal Res. Commun. 1, 12. doi: 10.1101/2021.01.05.425390

[B109] Zhang Y. Du H. Xu F. Ding Y. Gui Y. Zhang J.. (2020). Root-bacteria associations boost rhizosheath formation in moderately dry soil through ethylene responses. Plant Physiol. 183, 780–792. doi: 10.1104/pp.19.01020 32220965 PMC7271771

[B110] Zhang Q. Feng Y. X. Lin Y. J. Yu X. Z. (2022). Mathematical quantification of interactive complexity of transcription factors involved in proline-mediated regulative strategies in *Oryza sativa* under chromium stress. Plant Physiol. Biochem. 182, 36–44. doi: 10.1016/j.plaphy.2022.04.005 35460933

[B111] Zia R. Nawaz M. S. Yousaf S. Amin I. Hakim S. Mirza M.. (2021). Seed inoculation of desert-plant growth-promoting rhizobacteria induce biochemical alterations and develop resistance against water stress in wheat. Physiol. Plant 172, 990–1006. doi: 10.1111/ppl.13362 33547812

[B112] Zouari M. Hassena A. B. Trabelsi L. Rouina B. B. Decou R. Labrousse P. (2019). “Exogenous proline-mediated abiotic stress tolerance in plants: possible mechanisms,” in Osmoprotectant-mediated abiotic stress tolerance in plants. Eds. Hossain M. A. Kumar V. Burritt D. J. Fujita M. Mäkelä P. S. A. (Cham: Springer), 99–121. doi: 10.1007/978-3-030-27423-8_4

