# Supplementary figures and images for: Mitigation of water scarcity with sustained growth of Rice by plant growth promoting bacteria
Source: Front Plant Sci. 2023 Jan 23;14:1081537. doi: 10.3389/fpls.2023.1081537 (PMC9900138; doi:10.3389/fpls.2023.1081537)

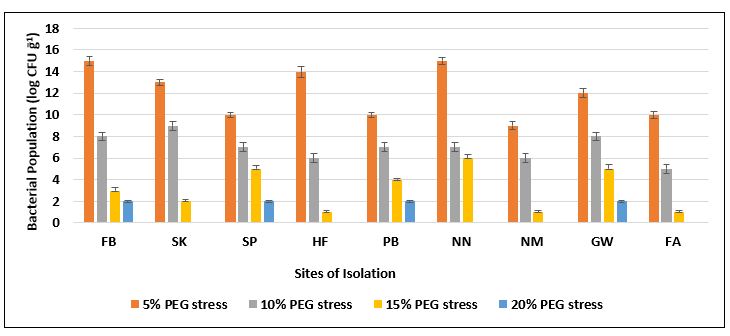

Supplement: Supplementary Figure 1 — Total colony forming units (CFU) obtained at different concentrations of Polyethylene Glycol (PEG)-induced stress in LB medium for isolation of drought tolerant bacteria. FB: Faisalabad, SK: Sialkot, SP: Sheikhupura, HF: Hafizabad, PB: Pindi Bhattian, NN: Nankana Sahib, NM: Narang Mandi, GW: Gujranwala and FA: Farooqabad. [file Image_1.jpeg]

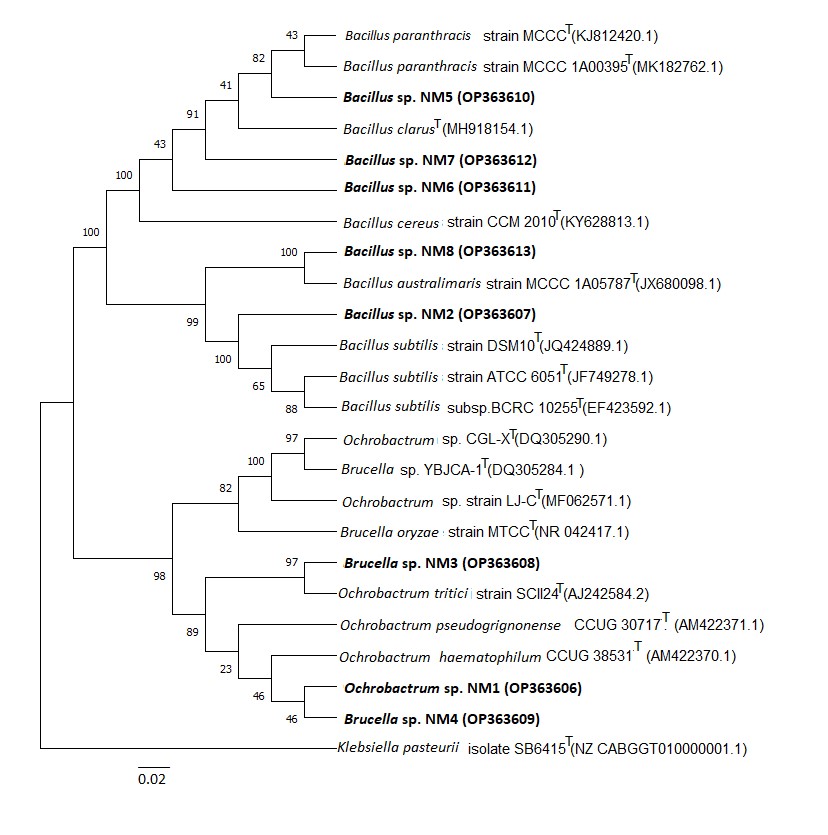

Supplement: Supplementary Figure 2 — Phylogenetic tree based on 16S rRNA sequencing of drought-tolerant bacteria [file Image_2.jpeg]

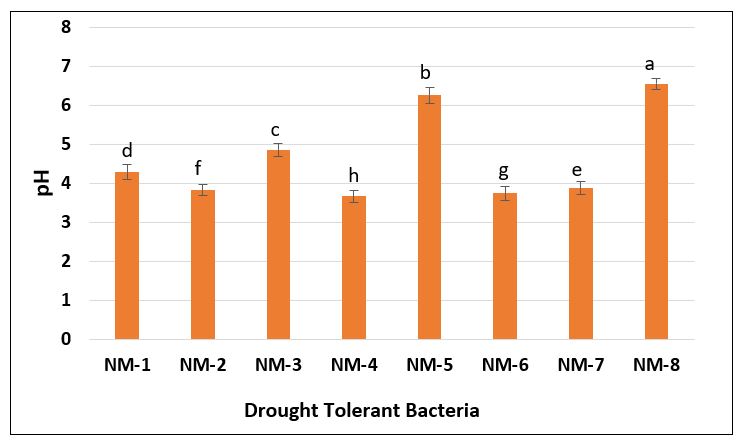

Supplement: Supplementary Figure 3 — pH change at 7th day of post inoculation (DPI) by rice rhizosphere drought tolerant bacteria in NBRIP′s broth having tricalcium phosphate (TCP) as insoluble phosphate source. [file Image_3.jpeg]
