# Supplementary material for: Mitigation of water scarcity with sustained growth of Rice by plant growth promoting bacteria
Source: Front Plant Sci. 2023 Jan 23;14:1081537. doi: 10.3389/fpls.2023.1081537 (PMC9900138; doi:10.3389/fpls.2023.1081537)
Supplement: Supplementary file 4 [file DataSheet_1.pdf]

## Table S1: Morpho-physiological characterization and 16S rRNA based identification of drought tolerant PGP bacteria

| S. No | Drought tolerant bacteria | Site of Isolation | Drought tolerance level | Colony Picture | Colony Morphology | Motility | Gram staining | Accession No. |
|---|---|---|---|---|---|---|---|---|
| 1 | *Ochrobactrum soli* NM-1 | Nankana Sahib | 20% | 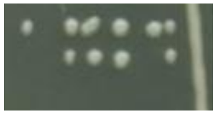 | Small, creamy-white, shiny with entire margins | + | - | OP363606 |
| 2 | *Bacillus subtilis* NM-2 | Faisalabad | 20% | 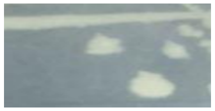 | Dry, smooth, white, flat with irregular margins | + | + | OP363607 |
| 3 | *Brucella anthropic* NM-3 | Pindi Bhattian | 20% | 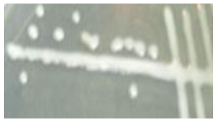 | Small, smooth, shiny, lobate, raised, white | + | - | OP363608 |
| 4 | *Brucella haematophilum* NM-4 | Gujranwala | 20% | 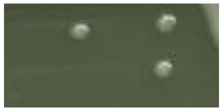 | shiny, medium, smooth, circular, convex, off-white with entire margins | + | - | OP363609 |
| 5 | *Bacillus* sp. NM-5 | Faisalabad | 20% | 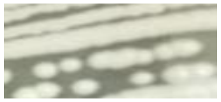 | Large, undulate, raised, shiny-with irregular margins | + | + | OP363610 |
| 6 | *Bacillus cereus* NM-6 | Sheikhupura | 20% | 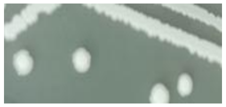 | Medium, smooth, shiny, circular, convex, white with entire margins | + | + | OP363611 |
| 7 | *Bacillus clarus* NM-7 | Kala Shah Kaku | 20% | 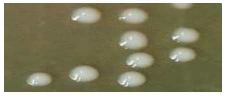 | Large, circular, white, entire, spread, convex, shiny with entire margins | + | + | OP363612 |
| 8 | *Bacillus australimaris* NM-8 | Sheikhupura | 20% | 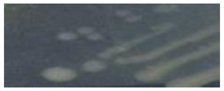 | Medium, off-white, undulate, slightly raised, smooth with irregular margins | + | + | OP363613 |

**Table S2: Effect of drought tolerant bacteria on seed germination and vigor index of different rice genotypes at 20% PEG-mediated osmotic stress in a plate assay under growth room conditions**

| | | V1 | | V2 | | V3 | |
|---|---|---|---|---|---|---|---|
| **Treatments** | **Strain name** | **% Germination** | **Seedling vigor index** | **% Germination** | **Seedling vigor index** | **% Germination** | **Seedling vigor index** |
| **Control** | | 100±6.00a | 1246.7±56c | 100±5.50a | 1263.3±60b | 100±6.52a | 1251.7±58bc |
| **Inoculated Control** | NM-1 | 90±4.50c | 1257.0±50c | 90±4.35c | 1272.0±56b | 95±4.00ab | 1280.3±60b |
| | NM-2 | 100±5.00a | 1508.3±65a | 100±4.5a | 1515.0±65a | 100±5.50a | 1621.7±67a |
| | NM-3 | 95±5.00b | 1293.7±57bc | 90±3.75c | 1273.5±50b | 90±4.00bc | 1264.5±62b |
| | NM-4 | 100±6.50a | 1525.0±60a | 100±6.00a | 1531.7±71a | 100±4.75a | 1626.7±65a |
| | NM-5 | 90±4.00c | 1258.5±54c | 90±4.00c | 1269.0±53b | 90±3.75bc | 1273.5±59b |
| | NM-6 | 100±4.50a | 1488.3±63ab | 95±4.00b | 1499.7±59a | 100±6.00a | 1510.0±60a |
| | NM-7 | 90±4.00c | 1288.5±58bc | 85±3.25d | 1273.0±62b | 90±3.55bc | 1294.5±58b |
| | NM-8 | 90±4.50c | 1291.5±53bc | 90±4.50c | 1266.0±56b | 85±4.00cd | 1290.3±55b |
| | Consortium | 100±5.00a | 1558.3±54a | 100±4.47a | 1591.7±70a | 100±4.53a | 1710.0±68a |
| **Osmotic Stressed** | | 60±3.25h | 630.0±24f | 80±3.45e | 840.0±40c | 80±3.50d | 957.3±43d |
| **Inoculated osmotic stressed** | NM-1 | 75±3.00f | 824.5±29def | 85±3.75d | 934.2±38c | 85±4.00cd | 973.7±45d |
| | NM-2 | 80±3.50e | 893.3±41de | 90±5.00c | 985.5±46c | 95±5.00ab | 1000.7±48d |
| | NM-3 | 70±3.00g | 702.3±25ef | 80±4.50e | 805.3±32c | 80±3.75d | 969.3±43d |
| | NM-4 | 90±4.35c | 957.0±45d | 95±5.50b | 1010.8±41c | 100±5.52a | 1046.7±47cd |
| | NM-5 | 70±3.45g | 757.2±33def | 80±4.00e | 865.3±43c | 85±4.25cd | 979.2±48d |
| | NM-6 | 85±4.00d | 943.2±37d | 90±4.75c | 996.0±50c | 95±4.00ab | 1013.3±46d |
| | NM-7 | 75±4.00f | 768.8±35de | 85±5.35d | 870.8±39c | 90±4.35bc | 963.0±47d |
| | NM-8 | 70±3.50g | 703.5±29ef | 80±4.00e | 858.7±42c | 85±3.55cd | 961.5±44d |
| | Consortium | 95±3.00a | 1009.2±35a | 100±4.50a | 1083.3±47a | 100±4.65a | 1100±53a |

Effect of drought tolerant bacterial inoculation individually and in consortium on percent germination and seedling vigor index of rice genotypes; **V1:** Super Basmati, **V2:** NIBGE-DT02 and **V3:** IR55419-04. Seeds were soaked in each bacterial culture (1 x $10^8$ CFU $mL^{-1}$) and in consortium (1 x $10^8$ CFU $mL^{-1}$) separately for 30 minutes prior to set on filter paper in petri plates. Control seeds were dipped in non-inoculated LB broth. Treatments; **Control** (non-inoculated seeds + Hoagland solution), **Inoculated control** (inoculated seeds + Hoagland solution), **Osmotic stressed** (non-inoculated seeds + 20% PEG-8000 supplemented Hoagland solution) and **inoculated osmotic stressed** (Inoculated seeds + 20% PEG-8000 supplemented Hoagland solution). Plates were incubated in growth room at

28±2°C for 7 days. Data were recorded at 7th DPI and all values are an average of three biological replicates per treatment. ± showed the standard deviation. Non-significant means are followed by same letter at p = 0.01 according to LSD.

## Table S3: Infrared thermal imaging to study the effect of inoculation on plant temperature during water stress under net house conditions

| Treatments | Genotypes | Temperature before stress | Temperature 5 DAS | Temperature 10 DAS | Temperature 15 DAS |
|---|---|---|---|---|---|
| **Non-inoculated Control** | V1 | 31.8±0.87a | 31.4±0.59cd | 31.58±0.78cd | 31.7±0.66d |
| | V2 | 31.9±0.73a | 31.6±0.26cd | 31.85±0.64cd | 32.0±0.87d |
| | V3 | 31.8±0.75a | 32.0±0.90c | 31.68±0.64cd | 31.9±0.47d |
| **Inoculated control** | V1 | 31.7±0.52a | 31.3±0.78cd | 31.45±0.78d | 31.6±0.54d |
| | V2 | 31.7±0.75a | 32.0±0.38c | 31.63±0.75cd | 31.9±0.82d |
| | V3 | 32.0±0.87a | 31.7±0.59cd | 31.92±0.78cd | 31.8±0.80d |
| **Inoculated stressed** | V1 | 31.6±0.57a | 32.9±0.73b | 34.07±0.94b | 35.0±0.64b |
| | V2 | 31.4±0.42a | 31.9±0.47c | 32.42±0.78c | 33.3±0.59c |
| | V3 | 31.9±0.64a | 31.1±0.54d | 32.03±0.61cd | 33.0±0.47c |
| **Non-inoculated stressed** | V1 | 31.8±0.49a | 34.0±0.94a | 35.67±0.52a | 37.3±0.47a |
| | V2 | 31.9±0.26a | 33.0±0.85b | 33.92±0.35b | 34.7±0.61b |
| | V3 | 31.6±0.57a | 32.9±0.40b | 33.63±0.71b | 34.2±0.82b |

Evaluation of drought tolerant PGPR consortium on plant temperature using Infrared thermal imaging (IRTI) in pot experiment under net house conditions. Rice genotypes; **V1:** Super Basmati, **V2:** NIBGE-DT02 and **V3:** IR55419-04. Treatments; Treatments: **non-inoculated control**, **inoculated control**, **inoculated stressed** and **non-inoculated stressed. DAS:** days after water stress. Data was averaged for six biological replicates. ± represent standard deviation. Means data was represented and means with same letter differ non-significantly while different letter showed data was significant at p=0.05 according to LSD.

**Table S4: SPAD (Soil and Plant Analyzer Development) to study the effect of inoculation on plant chlorophyll content during water stress under net house conditions**

| Treatments | Genotypes | Before stress | 5 DAS | 10 DAS | 15 DAS |
|---|---|---|---|---|---|
| **Non-inoculated Control** | V1 | 84.38±0.42cd | 84.07±0.71b | 84.00±0.77b | 84.17±2.53b |
| | V2 | 84.16±0.59d | 84.04±0.45b | 84.19±1.03b | 84.17±2.66b |
| | V3 | 84.07±0.65d | 84.15±0.45b | 84.04±1.13b | 84.24±3.80b |
| **Inoculated control** | V1 | 86.37±0.70abc | 86.44±0.78a | 86.15±0.64a | 86.19±3.56a |
| | V2 | 86.82±1.27a | 86.44±0.96a | 86.70±0.83a | 86.85±1.02a |
| | V3 | 86.60±0.92ab | 86.30±0.71a | 86.52±0.51a | 86.65±2.88a |
| **Inoculated stressed** | V1 | 84.15±3.04d | 84.04±3.18b | 81.89±2.82c | 80.82±2.36d |
| | V2 | 84.70±3.45bcd | 84.05±3.41b | 83.70±4.17b | 83.40±3.69bc |
| | V3 | 85.01±4.18abcd | 84.54±3.72b | 84.32±3.71b | 84.00±3.81b |
| **Non-inoculated stressed** | V1 | 84.54±3.86bcd | 81.54±2.67c | 80.70±3.07c | 77.61±2.56e |
| | V2 | 85.07±3.93abcd | 84.54±4.19ab | 81.87±2.82c | 80.80±3.33d |
| | V3 | 85.56±4.22abcd | 84.6±4.24b | 83.61±3.45b | 81.87±3.46cd |

Evaluation of drought tolerant PGPR consortium on plant chlorophyll content using SPAD meter in pot experiment under net house conditions. Rice genotypes; **V1:** Super Basmati, **V2:** NIBGE-DT02 and **V3:** IR55419-04. Treatments; Treatments: **non-inoculated control**, **inoculated control**, **inoculated stressed** and **non-**

**inoculated stressed**. **DAS:** days after water stress. Data was averaged for six biological replicates. ± represent standard deviation. Means data was represented and means with same letter differ non-significantly while different letter showed data was significant at p=0.05 according to LSD.

**Table S5: Determination of chlorophyll contents in response to bacterial inoculation during water stress in a pot experiment under net house conditions**

| Treatments | Genotypes | CHL a[a] | CHL b[b] | CHL t[c] |
|---|---|---|---|---|
| **Non-inoculated Control** | V1 | 26.06±0.55e | 15.13±0.73e | 41.52±1.28f |
| | V2 | 28.12±0.38d | 17.68±0.48d | 46.15±0.86e |
| | V3 | 33.39±0.22a | 29.33±0.54a | 63.11±0.76a |
| **Inoculated control** | V1 | 32.79±0.56ab | 23.22±0.14c | 56.42±0.71c |
| | V2 | 31.37±0.85c | 18.53±0.11d | 50.30±0.97d |
| | V3 | 31.89±0.50bc | 27.36±0.65b | 59.63±0.14b |
| **Inoculated stressed** | V1 | 21.04±0.38g | 15.67±0.45e | 36.97±0.83g |
| | V2 | 23.20±0.83f | 13.75±0.42f | 37.25±1.26g |
| | V3 | 23.36±0.69f | 13.97±0.10f | 37.63±0.60g |
| **Non-inoculated stressed** | V1 | 15.02±0.72i | 10.07±0.21h | 25.27±0.52j |
| | V2 | 18.76±0.90h | 12.58±0.31g | 31.57±1.22i |
| | V3 | 20.80±0.69g | 12.92±0.93fg | 33.99±1.63h |

Evaluation of drought tolerant PGPR consortium on plant chlorophyll a, b and t using biochemical analysis in a pot experiment under net house conditions. Rice genotypes; **V1:** Super Basmati, **V2:** NIBGE-DT02 and **V3:** IR55419-04. Treatments; **non-inoculated control**, **inoculated control**, **inoculated stressed** and **non-inoculated stressed**. [a]**-CHL a**: chlorophyll a (mg/g FW), [b]- CHL b: chlorophyll b (mg/g FW) and [c]**- CHL t:** chlorophyll t (mg/g FW). Data was averaged for six biological replicates. ± indicates standard deviation (SD). Means data was represented and means with same letter differ non-significantly while different letter showed data was significant at p=0.05 according to LSD.
